# Interference of iron deficiency in glycated hemoglobin testing and its clinical implications: a systematic review and meta-analysis

**DOI:** 10.11613/BM.2026.030503

**Published:** 2026-10-15

**Authors:** Emanuele Maria Coimbra Souza, Caroline Pereira Domingueti

**Affiliations:** Federal University of São João del-Rei, West Center Campus, Divinópolis, Brazil

**Keywords:** anemia, iron-deficiency, glycated hemoglobin, clinical laboratory techniques

## Abstract

**Introduction:**

Concentration of glycated hemoglobin (HbA1c) may be influenced by different types of anemia. This systematic review and meta-analysis aimed to evaluate the effect of iron deficiency anemia (IDA) and latent iron deficiency (LID) on HbA1c concentrations.

**Materials and methods:**

A search was conducted in Medline/PubMed, Web of Science, Embase, and LILACS up to May 2026. Eligible studies included cross-sectional, cohort, case-control, non-randomized intervention studies and clinical trials assessing the impact of IDA or LID on HbA1c concentrations. Methodological quality was evaluated using the ROBINS-I V2 tool for intervention studies and the Newcastle-Ottawa Scale for cross-sectional studies. Meta-analyses were performed using Review Manager 5 (The Cochrane Collaboration, Copenhagen, Denmark).

**Results:**

Among 43 studies evaluating IDA, 33 reported falsely elevated HbA1c concentrations. Of six studies assessing LID, two observed increased HbA1c concentrations. All four studies evaluating iron deficiency regardless of anemia status found elevated HbA1c. Among 19 intervention studies, 14 showed a reduction in HbA1c following iron supplementation. Meta-analyses including 30 studies confirmed increased HbA1c concentrations in patients with IDA (0.66% (0.31 to 1.00) or 7.21 mmol/mol (3.39 to 10.93), P < 0.001). Meta-analysis of four studies verified increased HbA1c in individuals with LID (0.66% (0.01 to 1.32) or 7.21 mmol/mol (0.11 to 14.43), P = 0.05). Meta-analysis of 14 intervention studies demonstrated a reduction in HbA1c following iron therapy (- 0.50% (- 0.92 to - 0.09) or 5.46 mmol/mol (- 10.05 to - 0.98), P = 0.02). Heterogeneity was mainly related to HbA1c assay methods and diagnostic criteria for anemia and iron deficiency.

**Conclusions:**

Iron deficiency anemia interferes with HbA1c measurement, leading to falsely elevated values, while LID may also increase HbA1c concentrations.

## Graphical abstract

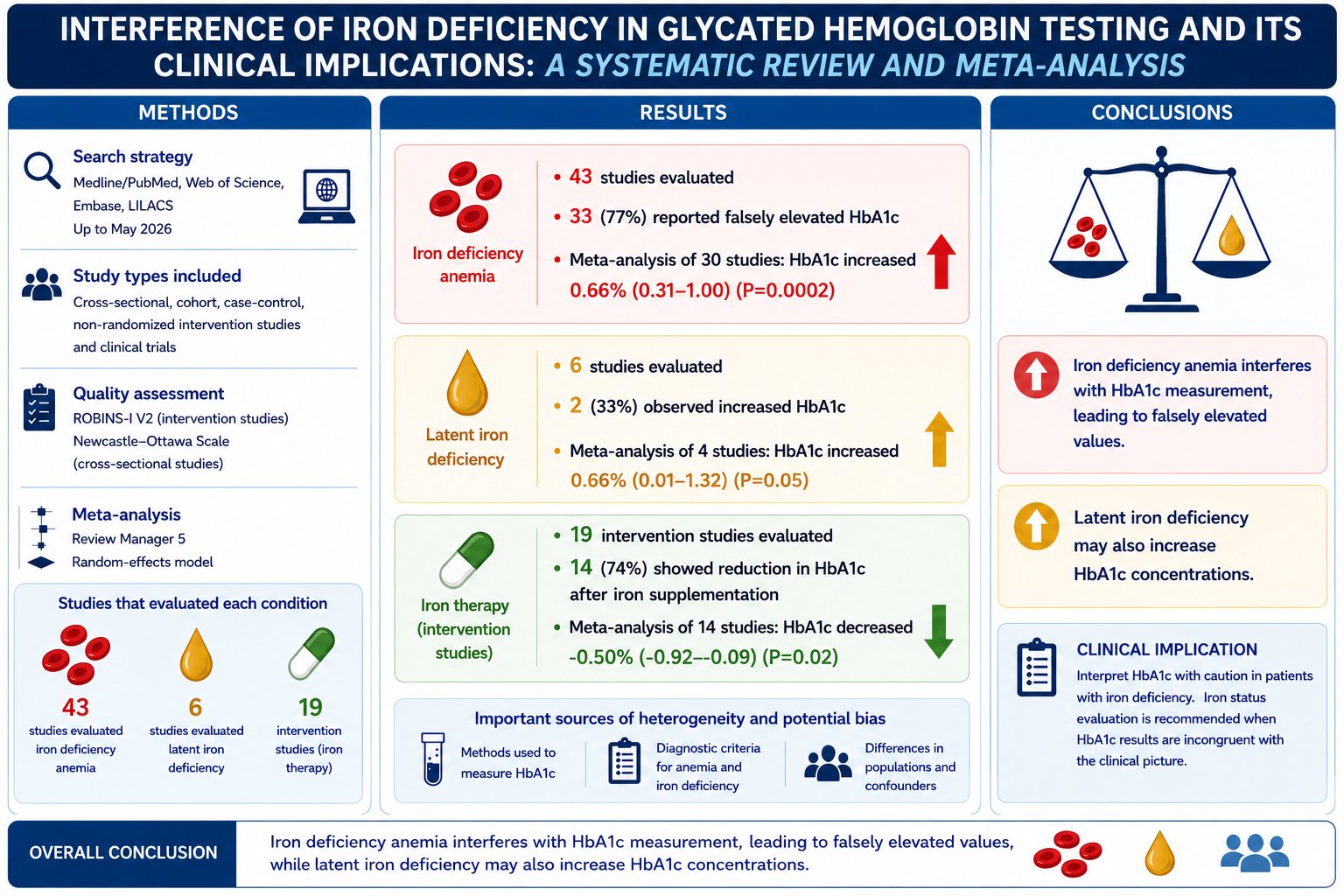

## Highlights

• There is a false increase in glycated hemoglobin (HbA1c) concentrations in patients with iron deficiency anemia

• There is a false increase in HbA1c concentrations in patients with latent iron deficiency

• Iron deficiency anemia and latent iron deficiency interfere with the HbA1c test

## Introduction

According to the World Health Organization (WHO), anemia is defined by low hemoglobin concentration in the bloodstream (*1*). Gardner *et al.* 2020 estimated that the global prevalence of anemia in 2019, considering all age groups, was 22.8%, equivalent to 1.74 billion people, with children under five being the most affected group, and that iron deficiency was the most common cause of anemia (*2*). Iron deficiency anemia (IDA) may result from gastrointestinal bleeding and menstruation in women, as well as from inadequate dietary iron intake and impaired iron absorption (*3*).

Laboratory diagnosis of IDA is made through hematological and biochemical tests, and there are three stages of iron deficiency. The first is iron depletion, which consists of a decrease in the body’s reserves. The best method for identifying this stage is a serum ferritin test, which is responsible for iron storage in the body. In the second stage, iron deficiency occurs, leading to iron-deficient erythropoiesis generated by the reduction of circulating minerals, resulting in decreased production of iron components and hemoglobin, but without decreasing their concentration. The tests that may be requested at this stage are serum iron and transferrin saturation. Finally, there is IDA, characterized by reduced hemoglobin concentrations that can be observed in the blood count (*4*).

There is substantial evidence indicating that IDA can interfere with glycated hemoglobin (HbA1c) measurements, leading to falsely elevated results (*5*). This effect is primarily attributed to the prolonged lifespan of erythrocytes observed in IDA, which increases their exposure time to circulating glucose and consequently enhances hemoglobin glycation. In addition, iron deficiency may alter the structure of hemoglobin and promote oxidative processes, such as hemoglobin peroxidation, further accelerating non-enzymatic glycation (*6*). This falsely elevated HbA1c could suggest poor glycemic control for patients with diabetes mellitus (DM) or lead to an incorrect diagnosis. Some studies have demonstrated that the clinical use of HbA1c, especially for diagnosis, should be associated with a complete blood count and markers of iron metabolism to assess whether the increase was being induced by iron deficiency (*7*, *8*).

In this context, it is important to conduct a systematic review and meta-analysis to carefully assess the interference of latent iron deficiency (LID) and IDA on HbA1c concentrations and the real impact of this deficiency on the interpretation of HbA1c test results for the diagnosis of DM and for monitoring glycemic control in patients with DM. Therefore, the PICOS-based research question is whether patients with IDA or LID exhibit higher HbA1c concentrations compared to those without iron deficiency.

## Materials and methods

Systematic review and meta-analysis conducted in accordance with the Preferred Reporting Items for Systematic reviews and Meta-Analyses (PRISMA) recommendations (*9*). This systematic review was not registered in the international PROSPERO database.

Selection of articles in Medline/PubMed, Web of Science, Embase, and LILACS using the descriptors “glycated hemoglobin” and their respective entry terms, in combination with the descriptors “anemia, iron- deficiency” and “iron deficiencies” and their respective entry terms using “AND” between the terms (Appendix) until May 2, 2026.

The eligibility criteria were established in accordance with the PRISMA recommendation and consist of observational cross-sectional, cohort, case-control, clinical trial or non-randomized intervention studies that evaluate the interference of LID or IDA in HbA1c testing (*9*).

Only studies whose experimental design allowed for the distinction of the following points, according to the acronyms PECOS (Table 1) and PICOS (Table 2), were included in the systematic review.

**Table 1:** PECOS strategy for study selection in the systematic review

**Patients**	**Patients with LID or IDA**
Exposure	Iron deficiency
Control	Patients without LID or IDA
Outcome	Interference in the HbA1c test
Study design	Cross-sectional observational, cohort, or case-control
LID - latent iron deficiency. IDA - iron deficiency anemia. HbA1c - glycated hemoglobin.	

**Table 2:** PICOS strategy for study selection in the systematic review

**Patients**	**Patients with LID or IDA**
Intervention	Iron supplementation
Control	Patients with LID or IDA before intervention
Outcome	Interference in the HbA1c test
Study design	Clinical trial or non-randomized intervention study
LID - latent iron deficiency. IDA - iron deficiency anemia. HbA1c - glycated hemoglobin.	

We also included studies comparing patients with different severities of IDA (severe, moderate and mild IDA). Studies that were not cross-sectional observational, cohort, case-control, non-randomized intervention or clinical trial were excluded, including systematic reviews, narrative reviews, conference abstracts, letters, editorials, case reports, notes, meta-analyses, and preclinical studies. Studies that did not assess at least one marker of iron metabolism (ferritin, transferrin saturation, serum iron, or total iron-binding capacity (TIBC)) to define IDA or LID were also excluded. In addition, studies evaluating the association between elevated ferritin concentrations and HbA1c were excluded. Articles published in Russian were also excluded.

The selection of studies was performed in two stages, both of which were carried out independently by two people. In the first stage, duplicate articles were excluded, and then a preliminary reading of the title and abstract of the articles was performed with the aim of including only those that are observational cross-sectional, cohort, case-control, clinical trials or non-randomized intervention studies and that assessed the interference of LID or IDA in the HbA1c test. In the second stage, the preselected articles were read in full to assess their inclusion in the study according to the eligibility criteria. A flowchart was then constructed summarizing the number of articles included and excluded in each stage according to the established criteria, in accordance with the PRISMA recommendation (*9*).

The following data were extracted from the selected articles to construct tables: author, year of publication, country, study design, administered iron dose, method used to measure HbA1c, main exclusion criteria, diagnostic criteria for anemia, biomarkers/diagnostic criteria for iron deficiency, sample size, patient characteristics (gender, age group, presence or absence of diabetes mellitus, *etc.*), main results, effect of iron supplementation on HbA1c, interference of IDA or LID in HbA1c testing, interference in the risk of classification as prediabetes or diabetes mellitus by HbA1c testing.

The methodological quality of the non-randomized studies included in the systematic review was assessed using the ROBINS-I V2 tool (*10*). This tool assesses the following domains of bias: confounding, classification of interventions, selection of participants into the study, missing data, measurement of outcomes, selection of the reported result. An overall risk of bias judgment was formulated based on the weakest component principle: the overall risk followed the ranking of the domain with the highest level of bias identified. Studies were categorized as having low, moderate, serious or critical risk.

The methodological quality of the cross-sectional studies included in this systematic review was assessed using the Newcastle-Ottawa Scale (NOS) adapted for cross-sectional studies (*11*). According to the NOS, a maximum of nine stars can be awarded to each study. One star could be assigned for each of the following criteria: representativeness of the sample, selection of controls, sample size, diagnosis, and statistical test. Up to two stars could be awarded for comparability and for the accuracy of the method used to measure HbA1c concentrations. Studies were classified as low quality if they received up to three stars, as moderate quality if they received four to six stars, and as high quality if they received seven or more stars.

### Statistical analysis

Meta-analyses were performed on studies that: (i) compared HbA1c concentrations before and after iron supplementation; (ii) compared HbA1c concentrations between patients with and without IDA; and (iii) compared HbA1c concentrations between patients with and without LID. Studies that did not report mean values, standard deviations, or sample sizes were excluded from the meta-analyses. Studies were also excluded if they did not include an appropriate control group.

The mean, standard deviation, and sample size before and after iron supplementation, or of patients with and without IDA or patients with and without LID in each study were used in the meta-analyses, and the difference between the means was calculated, P ≤ 0.05 was considered statistically significant. Some studies reported results exclusively as subclassifications; therefore, overall combined mean and standard deviation estimates were calculated to enable their inclusion in the analysis. Heterogeneity between studies was assessed using the I^2^ test, with studies presenting I^2^ > 50% and P < 0.10 being considered heterogeneous. Fixed-effect and random-effect models were used in the meta-analysis calculations in the presence of homogeneity and heterogeneity, respectively. The statistical program Review Manager (RevMan) version 5 (The Cochrane Collaboration, Copenhagen, Denmark) was used to perform the meta-analyses.

Subgroup analyses were performed to explore potential sources of heterogeneity among the included studies and to assess the consistency of the results across different clinical and methodological contexts. The predefined subgroups included sex (studies including both sexes, those restricted to nonpregnant women, and those including pregnant women), age group (children and adolescents *vs.* adults), and diabetes status (presence of DM *vs.* exclusion of patients with DM). Additional subgroup analyses considered studies excluding patients with chronic kidney disease (CKD), those excluding individuals with inflammation and/or elevated C-reactive protein (CRP) and those excluding patients with anemia not caused by iron deficiency. Methodological subgroups were defined according to the laboratory method used for HbA1c measurement (high-performance liquid chromatography (HPLC) *vs.* immunoturbidimetry), the criteria used to define anemia according to the WHO, and the criteria used to define iron deficiency according to the WHO (*12*, *13*). Publication bias was assessed by visual inspection of funnel plots.

## Results

Figure 1 shows the flowchart of article selection for this systematic review. After evaluating the eligibility criteria, 61 articles were included in the systematic review. Table 3 shows the location, study design, main exclusion criteria, HbA1c measurement method, diagnostic criteria for anemia and biomarkers/diagnostic criteria for iron deficiency in the studies included in the systematic review.

**Figure 1:**
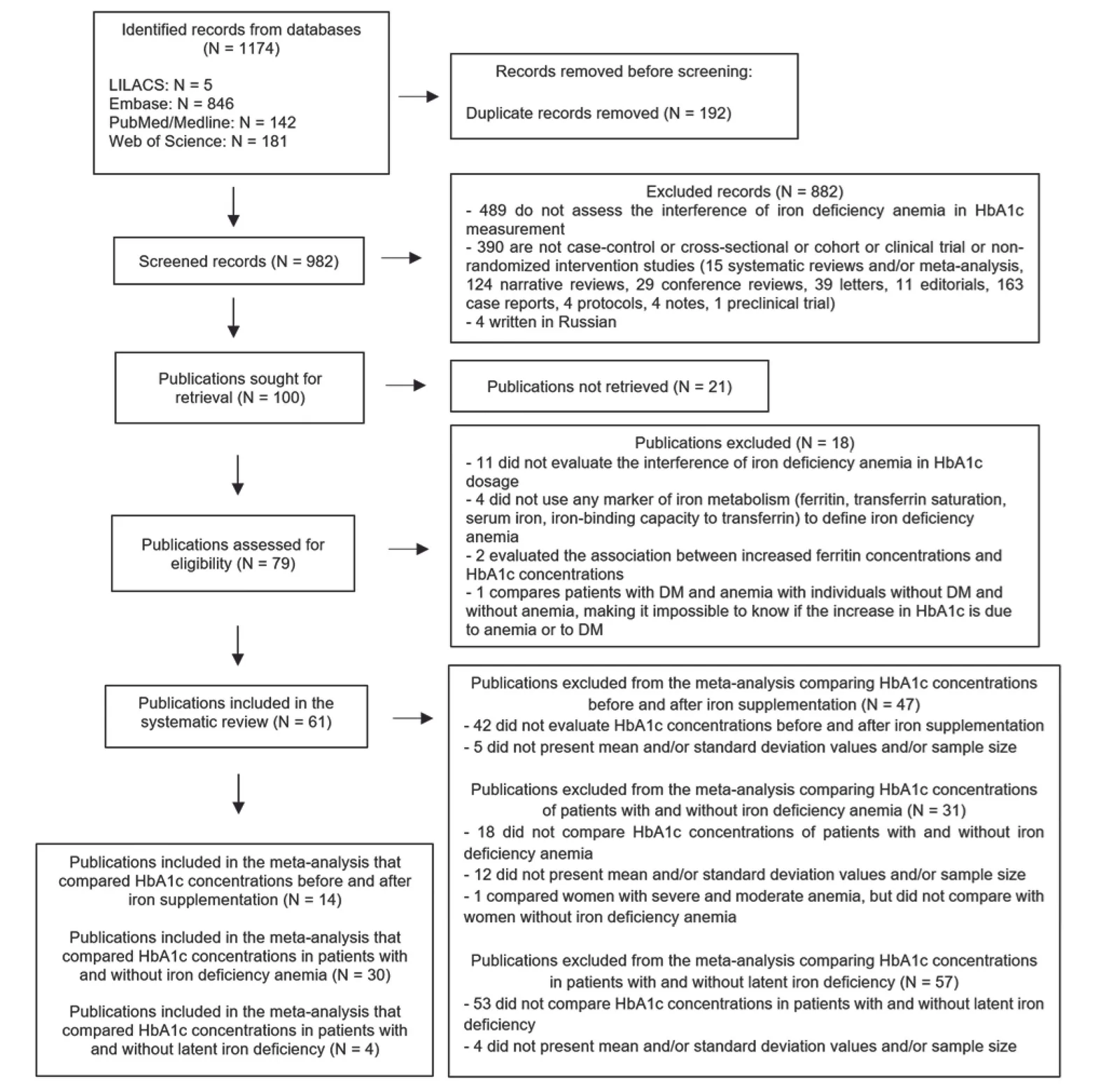
Flowchart of the selection of articles evaluating the interference of iron deficiency in glycated hemoglobin (HbA1c) measurement that were included in the systematic review

**Table 3:** Location, study design, main exclusion criteria, HbA1c measurement method, diagnostic criteria for anemia and biomarkers/diagnostic criteria for iron deficiency in the studies included in the systematic review

**Author, year**	**Country**	**Study design/ Iron dose** **administered**	**Main exclusion criteria**	**HbA1c measurement method**	**Diagnostic criteria for anemia**	**Biomarkers/diagnostic criteria for iron deficiency**
Mukasa *et al*., 2026 (*14*)	Uganda	Cross-sectional/ NA	Anemia, hemoglobinopathies, CKD	Enzymatic method	NI	Ferritin < 70 μg/L
Tedong *et al*., 2026 (*15*)	Cameroon	Cross-sectional/ NA	Hemolytic anemia, hemoglobinopathies, G6PD deficiency, thalassemia, malaria, infections, inflammation, elevated CRP concentrations	Immuno- turbidimetry	Hb < 130 g/L (M) or < 120 g/L (F)	Ferritin < 30 μg/L (M), ferritin < 15 μg/L (F), serum iron, TIBC
Deepali *et al*., 2025 (*16*)	India	Cross-sectional/ NA	DM, hemoglobinopathies, recent blood transfusion, severe non-IDA anemia, CKD	HPLC	NI	Ferritin, serum iron, TIBC
Koga and Ishibashi, 2025 (*17*)	Japan	Cross-sectional/ NA	NI	HPLC and enzymatic method	Hb < 80 g/L	Ferritin, serum iron, TIBC
Dutta *et al*., 2024 (*18*)	India	Cross-sectional/ NA	DM, hemolytic anemia, hemoglobinopathies, acute blood loss, vitamin B12/folate deficiency, treatment with erythropoietin, CKD	Latex agglutination	Hb < 130 g/L (M) or < 120 g/L (F)	Ferritin < 15 µg/L, serum iron > 30 µg/dL, TIBC > 360 µg/dL, transferrin saturation < 15%
Firat *et al*., 2024 (*19*)	Turkey	Cross-sectional/ NA	Hemoglobinopathies, vitamin B12/folate deficiency, chronic inflammatory diseases, elevated CRP concentrations	HPLC	Hb < 110 g/L	Ferritin < 15 μg/L
Haseena *et al*., 2024 (*20*)	India	Cross-sectional/ NA	DM, Anemia not caused by iron deficiency, hemoglobinopathies, vitamin B12/folate deficiency, acute blood loss, CKD	Immuno- turbidimetry	NI	Ferritin
Kanojia *et al*., 2024 (*21*)	India	Non-randomized intervention/ NI	DM, hemolytic anemia, hemoglobinopathies	NI	NI	Ferritin
Kesharwani *et al*., 2024 (*22*)	India	Cross-sectional/ NA	DM, hemoglobinopathies, blood transfusion, CKD	HPLC	Hb < 130 g/L (M) or < 120 g/L (F)	Ferritin < 30 μg/L, serum iron, TIBC
Krishnan *et al*., 2024 (*23*)	India	Cross-sectional/ NA	DM, acute blood loss, hemolytic anemia, hemoglobinopathies	Immuno- turbidimetry	Hb < 130 g/L (M) or < 120 g/L (F)	Ferritin < 15 µg/L
Kumar *et al*., 2024 (*24*)	India	Cross-sectional/ NA	DM, anemia not caused by iron deficiency, sickle cell disease, thalassemia, prolonged blood loss, CKD	Cation exchange chromatography	Hb < 130 g/L (M) or < 120 g/L (F)	Ferritin, serum iron
Nakum *et al*., 2024 (*25*)	India	Cross-sectional/ NA	NI	NI	NI	Ferritin < 22 µg/L (M), ferritin < 10 µg/L (F), serum iron, TIBC
Patel *et al*., 2024 (*26*)	India	Non-randomized intervention/ 100 mg/day of iron for 3 months	DM	NI	NI	Ferritin
Suvethasri *et al*., 2024 (*27*)	India	Cross-sectional/ NA	DM, hemoglobinopathies, hemolytic anemia, CKD	NI	Hb < 100 g/L	Ferritin
Shubham *et al*., 2024 (*28*)	India	Non-randomized intervention/ 160 mg/day of ferric ammonium citrate for 3 months	DM, hemolytic anemia, hemoglobinopathies, acute or chronic blood loss, recent blood transfusion, CKD	NI	Hb < 130 g/L (M) or < 120 g/L (F)	Ferritin
Taati *et al*., 2024 (*29*)	Iran	Clinical trial/ 100 mg/day of ferrous sulfate for 8 or 16 weeks	Anemia not caused by iron deficiency	NI	Hb < 130 g/L (M) or < 120 g/L (F)	Ferritin
Thakker *et al*., 2024 (*30*)	India	Non-randomized intervention/ 100 mg/day of iron for 4 weeks	DM, bleeding disorders, CKD	Immuno- turbidimetry	Hb < 110 g/L	Serum iron, TIBC, transferrin saturation
Alzahani *et al.,* 2023 (*31*)	Saudi Arabia	Non-randomized intervention/ NI	Anemia not caused by iron deficiency, recent blood loss, recent blood transfusion, CKD	HPLC	Hb < 130 g/L (M) or < 120 g/L (F)	Ferritin < 30 µg/L
Elsheikh *et al*., 2023 (*32*)	Egypt	Cross-sectional/ NA	Acute or chronic blood loss, hemolytic anemia, hemoglobinopathies, CKD	NI	Hb < 130 g/L (M) or < 120 g/L (F)	Ferritin < 15 µg/L
Gharde *et al*., 2023 (*33*)	India	Cross-sectional/ NA	DM, anemia not caused by iron deficiency, sickle cell disease, thalassemia, acute or chronic blood loss, CKD	NI	Hb < 130 g/L (M) or < 120 g/L (F)	Ferritin, serum iron, TIBC
Kumar *et al.,* 2023 (34)	Pakistan	Cross-sectional/ NA	Acute blood loss, hemolytic anemia, hemoglobinopathies, CKD	HPLC	NI	Serum iron < 59 μg/dL, transferrin saturation < 15%
Nosheen *et* *al.,* 2023 (*35*)	Pakistan	Non-randomized intervention/ NI	NI	NI	Hb < 140 g/L (M) or < 120 g/L (F)	Ferritin, serum iron, TIBC
Aydin *et al*., 2022 (*36*)	Turkey	Non-randomized intervention/ 270 mg/day of ferrous sulfate for 3 months	Elevated CRP concentrations, anemia not caused by iron deficiency, recent blood transfusion, CKD	HPLC	Hb < 120 g/L	Ferritin < 15 μg/L
El-Agouza *et al.,* 2022 (*37*)	Palestine	Non-randomized intervention/ 325 mg/day of ferrous sulfate	NI	Cation exchange chromatography	NI	Ferritin
Estrella *et al*., 2022 (*38*)	United States	Cross-sectional/ NA	CKD	HPLC	Hb < 137 g/L (M 20 to 49 years) or < 133 g/L (M 50 to 69 years) or < 124 g/L (M ≥ 70 years) or < 120 g/L (F 20 to 69 years) or < 118 g/L (F ≥ 70 years)	Ferritin < 27 μg/L, serum iron < 10.7 μmol/L, TIBC > 77 μmol/L, transferrin saturation < 15%
Janice *et al*., 2022 (*39*)	India	Cross-sectional/ NA	Acute blood loss, CKD	Nephelometry	Hb < 100 g/L	Ferritin < 90 μg/L
Jyothsna *et al.,* 2022 (*40*)	India	Cross-sectional/ NA	DM, inflammatory disease, acute illness, anemia not caused by iron deficiency, CKD	HPLC	Hb < 137 g/L (M 20 to 49 years) or < 133 g/L (M 50 to 69 years) or < 124 g/L (M ≥ 70 years) or < 120 g/L (F 20 to 69 years) or < 118 g/L (F ≥ 70 years)	Ferritin < 12 µg/L, transferrin saturation < 15%
Pradeepa *et al.,* 2022 (*41*)	India	Cross-sectional/ NA	Acute or chronic blood loss, hemoglobinopathies	HPLC	LID: Hb ≥ 130 g/L (M) or ≥ 120 g/L (F), IDA: Hb < 130 g/L (M) or < 120 g/L (F)	Ferritin < 70 μg/L
Rao *et al.,* 2022 (42)	United States	Cross-sectional/ NA	NI	Enzymatic method	NI	Ferritin, serum iron, transferrin saturation, TIBC
Altuntas *et al*., 2021 (*43*)	Turkey	Non-randomized intervention/ 100 mg/day of iron for 3 months	DM, hemoglobinopathies, hemolytic anemia, CKD	HPLC	Hb < 120 g/L (F) or < 130 g/L (M)	Ferritin
Bindayel, 2021 (*44*)	Saudi Arabia	Cross-sectional/ NA	DM, anemia not caused by iron deficiency, CKD	HPLC	Hb ≤ 120 g/L	Ferritin < 15 μg/L
Kanchana and Pushpa, 2021 (*45*)	India	Cross-sectional/ NA	DM, hemolytic anemia, CKD	Immuno- turbidimetry	Hb < 110 g/L	Ferritin < 15 μg/L (M) or < 9 μg/L (F)
Lyons *et al.,* 2020 (46)	Netherlands	Cross-sectional/ NA	Hb variants HbAS and HbAC, CKD	HPLC	NI	Ferritin < 15 μg/L if CRP < 5 mg/mL or ferritin < 30 μg/L if CRP ≥ 5 mg/mL
Mahgoob and Moussa, 2020 (*47*)	Egypt	Cross-sectional/ NA	Anemia not caused by iron deficiency, recent blood loss, hemoglobinopathies	Colorimetric	NI	Ferritin < 12 μg/L (< 5 years) or < 15 μg/L (≥ 5 years)
Pilla *et al.,* 2020 (*48*)	India	Non-randomized intervention/ NI	DM, hemolytic anemia, aplastic anemia, anemia of chronic diseases	NI	Hb < 120 g/L (F) or < 130 g/L (M)	Ferritin, serum iron
Purbey *et al.,* 2020 (*49*)	India	Non-randomized intervention/ 160 mg/day of ferric ammonium citrate for 3 months	DM, hemoglobinopathies, hemolytic anemia, acute or chronic blood loss, recent blood transfusion, CKD	NI	Hb < 120 g/L (F) or < 130 g/L (M)	Ferritin
Sabitha *et al.,* 2020 (*50*)	India	Cross-sectional/ NA	Inflammatory disease	HPLC	NI	Serum iron < 50 ng/dL and transferrin saturation < 14%
Intra *et al.,* 2019 (*51*)	Italy	Cross-sectional/ NA	DM, infectious disease, hematological disease, blood transfusion, CKD	HPLC	Hb ≤ 130 g/L (M) or ≤ 110 g/L (F)	Ferritin ≤ 17 μg/L (M) or ≤ 10 μg/L (F)
Silva *et al.,* 2019 (*52*)	Brazil	Cross-sectional/ NA	DM	HPLC and Immuno- turbidimetry	Hb < 130 g/L (M) or < 120 g/L (F)	Ferritin < 15 μg/L
Wei *et al.,* 2019 (*53*)	China	Cross-sectional/ NA	NI	HPLC	NI	Quartile of transferrin and transferrin saturation concentrations
Akkermans *et al.,* 2018 (*54*)	Netherlands	Non-randomized intervention/ 9 mg/kg/day of ferrous fumarate for 2 months	Acute infection and/or inflammation (CRP ≥ 10 mg/L), hemoglobinopathies, recent blood transfusion	HPLC	IDA: Hb 2 standard deviations below the mean for children of the same age, LID: Hb normal	IDA/Absolute LID: Ferritin < 12 μg/L (< 5 years) or < 15 μg/L (≥ 5 years) Functional LID: zinc protoporphyrin > 61 μmol/mol heme (< 5 years) or 70 μmol/mol heme (≥ 5 years) or RDW-CV > 14% or RDW-SD > 43.39 fL
Eser *et al.,* 2018 (*55*)	Turkey	Non-randomized intervention/ NI	DM, hemoglobinopathies	Enzymatic method	Hb < 110 g/L in the first and third trimesters of pregnancy or < 105 g/L in the second trimester of pregnancy	Ferritin < 30 μg/L
Hashimoto and Koga, 2018 (*56*)	Japan	Cross- sectional/ NA	Elevated CRP concentrations, CKD	HPLC	NI	Ferritin < 15 μg/L
Intra *et al.,* 2018 (*57*)	Italy	Cross- sectional/ NA	DM, infectious disease, hematological disease, anemia not caused by iron deficiency, CKD	HPLC	Hb ≤ 130 g/L (M) or ≤ 115 g/L (F)	Ferritin ≤ 17 μg/L (M) or ≤ 10 μg/L (F)
Urrechaga, 2018 (*58*)	Spain	Cross- sectional/ NA	Acute phase reaction (CRP > 5.0 mg/L), macrocytic anemia	HPLC	IDA: Hb < 130 g/L (M) or < 120 g/L (F), LID: Hb ≥ 130 g/L (M) or ≥ 120 g/L (F)	Ferritin < 50 μg/L (M) or < 30 μg/L (F)
Madhu *et al.*, 2017 (*59*)	India	Non-randomized intervention/ 100 mg/day of Fe for 3 months	DM, chronic inflammatory disease (CRP > 0.6 mg/dL), hemolytic anemia, hemoglobinopathies, CKD	HPLC	Hb < 100 g/L	Ferritin ≤ 15 μg/L, serum iron ≤ 7.17 μmol/L, TIBC ≥ 71.6 μmol/L, transferrin saturation ≤ 16%
Nasli-Esfagani *et al.,* 2017 (*60*)	Iran	Clinical trial/ 200 mg/day of ferrous sulfate for 3 months	Hemoglobinopathies, treatment with erythropoietin, active infection, recent blood loss or blood transfusion, hemolytic anemia, CKD	HPLC	Hb < 130 g/L (M) or < 120 g/L (F)	Ferritin < 9 μg/L (M) or < 15 μg/L (F)
Rajagopal *et al.,* 2017 (*61*)	India	Cross-sectional/ NA	DM, hemolytic anemia, blood loss, CKD	HPLC	Hb < 130 g/L (M) or < 120 g/L (F)	Ferritin < 15 μg/L, serum iron < 60 μg/dL
Attard *et al.,* 2015 (*62*)	China	Cross-sectional/ NA	NI	HPLC	Hb < 130 g/L (M) or < 120 g/L (F)	Ferritin < 15 μg/L
Hong *et al.,* 2015 (*63*)	South Korea	Cross-sectional/ NA	NI	HPLC	Hb < 130 g/L (M) or < 120 g/L (F)	Ferritin < 15 μg/L, transferrin saturation < 10%
Christy *et al.,* 2014 (*64*)	India	Cross-sectional/ NA	DM, hemoglobinopathies, hemolytic anemia, CKD	HPLC	Hb < 120 g/L (M) or < 110 g/L (F)	Ferritin < 29 μg/L (M) or < 20 μg/L (F)
Shanthi *et al.*, 2013 (*8*)	India	Cross-sectional/ NA	DM, hemoglobinopathies, hemolytic anemia, CKD	Immuno- turbidimetry	Hb < 110 g/L	Ferritin < 15 μg/L (M) or < 9 μg/L (F)
Hardikar *et al.,* 2012 (*65*)	India	Cross-sectional/ NA	NI	HPLC	Hb < 130 g/L (M) or < 120 g/L (F)	Ferritin < 15 μg/L
Rafat *et al.,* 2012 (66)	India	Cross-sectional/ NA	DM, blood transfusion, acute blood loss, hemolytic anemia, Hb variants	HPLC	NI	Ferritin, serum iron, TIBC
Satriawibawa *et al.,* 2012 (*67*)	Indonesia	Cross-sectional/ NA	Chronic inflammation, chronic bleeding, hemolytic anemia, thalassemia, CKD	Immuno- turbidimetry	NI	Serum iron < 50 μg/dL
Sinha *et al.*, 2012 (*68*)	India	Non-randomized intervention/ NI	DM, acute blood loss, hemolytic anemia, hemoglobinopathies, CKD	Cation exchange chromatography	Hb < 130 g/L (M) or < 120 g/L (F)	Ferritin < 30 μg/L
Ford *et al.,* 2011 (*69*)	United States	Cross-sectional/ NA	NI	HPLC	Hb < 137 g/L (M 20 to 49 years) or < 133 g/L (M 50 to 69 years) or < 124 g/L (M ≥ 70 years) < 120 g/L (F 20 to 69 years) or < 118 g/L (F ≥ 70 years)	At least two of: ferritin < 12.1 μg/L, transferrin saturation < 15%, erythrocyte protoporphyrin > 1.24 μmol/L
Kim *et al.,* 2010 (*70*)	United States	Cross-sectional/ NA	CKD	HPLC	NI	At least two of: ferritin ≤ 15 µg/L, erythrocyte protoporphyrin > 70 µg/dL, transferrin saturation < 16%
Koga *et al.,* 2010 (*71*)	Japan	Cross-sectional/ NA	NI	HPLC	LID: Hb ≥ 114 g/L IDA: Hb < 114 g/L	Ferritin < 15 μg/L
Coban *et al.,* 2004 (*72*)	Turkey	Non-randomized intervention/ 100 mg/day of ferrous sulfate for 3 months	DM, hemoglobinopathies, hemolytic anemia, CKD	Immuno- turbidimetry	NI	Ferritin < 15 μg/L (M) or < 9 μg/L (F)
Tarim *et al.*, 1999 (*73*)	Turkey	Non-randomized intervention/ 6 mg/kg/day of oral Fe for 3 months	NI	HPLC	NI	Ferritin, serum iron, transferrin saturation, TIBC
HbA1c - glycated hemoglobin. CKD - chronic kidney disease. CRP - C-reactive protein. DM - diabetes mellitus. F - female. Fe - elemental iron. Hb - hemoglobin. HPLC - high performance liquid chromatography. ID - iron deficiency. IDA - iron deficiency anemia. LID - latent iron deficiency. M - male. NI - not informed. NA - not applicable. RDW-CV - red cell distribution width. CV - coefficient of variation. RDW-SD - red cell distribution width. SD - standard deviation. TIBC - total iron-binding capacity.						

Among the 61 studies included in the systematic review, most were cross-sectional (N = 42, 0.69), followed by non-randomized intervention studies (N = 17, 0.28), with only two clinical trials (N = 2, 0.03). The included studies were conducted across diverse geographic regions, with the highest representation from India (N = 27, 0.44), followed by Turkey (N = 6, 0.10), the United States (N = 4, 0.06), and Japan (N = 3, 0.05). Regarding the methods used for HbA1c measurement, most studies employed HPLC (N = 33, 0.54), immunoturbidimetry (N = 9, 0.15), enzymatic assays (N = 3, 0.05), and cation exchange chromatography (N = 3, 0.05).

The main exclusion criteria reported across studies included CKD (N = 34, 0.56), DM (N = 29, 0.48), hemoglobinopathies or hemoglobin variants, including thalassemia and sickle cell disease (N = 30, 0.49), hemolytic anemia (N = 21, 0.34), and acute or chronic blood loss (N = 17, 0.28), recent blood transfusion (N = 10, 0.16), non-IDA (N = 10, 0.16), inflammatory diseases (N = 7, 0.11), infectious or acute conditions (N = 6, 0.10), elevated CRP concentrations (N = 6, 0.10), macrocytic anemia or vitamin B12/folate deficiency (N = 4, 0.06). Notably, 12 studies (0.20) did not report exclusion criteria necessary to rule out anemia unrelated to iron deficiency or inflammation or DM.

The criteria used to diagnose anemia varied across studies. Twenty-four studies (0.39) applied the WHO definition, while 18 (0.30) used alternative criteria (*12*). Nineteen studies (0.31) did not report the diagnostic criteria. The biomarkers used to assess IDA and LID also varied across studies. Ferritin was the most frequently reported marker (N = 53, 0.87), followed by serum iron (N = 20, 0.33), TIBC (N = 14, 0.23), and transferrin saturation (N = 13, 0.21). The WHO criteria for defining iron deficiency based on ferritin concentrations were applied in 17 studies (0.28) (*13*).

The methodological quality of the intervention studies included in the systematic review, as assessed using the ROBINS-I V2 tool, is presented in Supplementary Table 1. Of the 19 studies, five were classified as having a moderate risk of bias, while 14 were classified as having a serious risk of bias.

The methodological quality of the cross-sectional studies, as assessed using the NOS adapted for cross-sectional studies, is presented in Supplementary Table 2. Of the 42 articles, 24 (0.57) received four to six stars and were therefore classified as having moderate quality, 17 (0.40) received one to three stars and were classified as having low quality, and only one (0.02) received seven or more stars and was classified as having high quality.

Table 4 shows sample size and characteristics of the patients, main results, and effect of iron supplementation on HbA1c concentrations in the studies included in the systematic review that assessed HbA1c concentrations before and after iron supplementation. Among the 19 studies, most included adults (15/19), two included pregnant women, one children and adolescents, and another adults and adolescents. Some studies included patients with type 1 diabetes mellitus (T1DM) (3/19) and type 2 diabetes mellitus (T2DM) (3/19). Overall, HbA1c concentrations decreased following iron supplementation in the majority of studies (14/19), increased in two studies - reflecting normalization of initially low HbA1c concentrations - and remained unchanged in three studies.

**Table 4:** Sample size and characteristics of patients, main results, and effect of iron supplementation on HbA1c concentrations in studies included in the systematic review that assessed HbA1c concentrations before and after iron supplementation

**Author, year**	**Sample size and characteristics of patients**	**HbA1c before iron supplementation**	**HbA1c after supplementation**	**P**	**Effect of iron supplementation** **on HbA1c test**
Kanojia *et al*., 2024 (*21*)	50 adults without DM with IDA	7.16 ± 0.88%	5.48 ± 0.31%	< 0.001	Decreased in adults without DM
Patel *et al*., 2024 (*26*)	92 adults without DM with IDA	4.63 ± 0.32%	5.82 ± 0.34%	< 0.001	Increased in adults without DM
Shubham *et al*., 2024 (*28*)	50 adults without DM with IDA	5.92 ± 0.37%	5.49 ± 0.42%	< 0.001	Decreased in adults without DM
Taati *et al*., 2024 (*29*)	27 adults with T2DM with IDA (16 weeks) 28 adults with T2DM with IDA (8 weeks)	8.123 ± 1.791% 7.122 ± 1.612%	7.989 ± 2.201% 7.311 ± 2.106%	> 0.05 > 0.05	No change in adults with T2DM
Thakker *et al*., 2024 (*30*)	155 pregnant women without DM with IDA	5.53 ± 0.48%	5.23 ± 0.44%	< 0.001	Decreased in pregnant women without DM
Alzahrani *et al.,* 2023 (*31*)	104 adults with IDA	5.75%	5.44%	< 0.001	Decreased in adults
Nosheen *et al.,* 2023 (*35*)	60 adults with T1DM with IDA (30 women and 30 men)	8.603% Women: 9.43% Men: 7.79%	7.608% Women: 8.09% Men: 7.12%	< 0.010 < 0.010 < 0.010	Decreased in adults (women and men) with T1DM
Aydin *et al*., 2022 (*36*)	146 adults with T2DM with IDA	7.09 ± 0.51%	6.69 ± 0.53%	< 0.001	Decreased in adults with T2DM
El-Agouza *et al.,* 2022 (*37*)	730 adults with IDA	6.15 ± 0.62%	5.25 ± 0.45%	< 0.001	Decreased in adults
Altuntas *et al*., 2021 (*43*)	131 adults without DM with IDA	5.4 ± 0.5%	5.5 ± 0.3%	0.057	No change in adults without DM
Pilla *et al.,* 2020 (*48*)	100 adults without DM with IDA (mild and moderate IDA x severe IDA)	Mild and moderate IDA: 6.1 ± 0.23% Severe IDA: 5.5 ± 0.24%	Mild and moderate IDA: 5.1 ± 0.14% Severe IDA: 4.6 ± 0.2%	< 0.001 < 0.001	Decreased in adults without DM
Purbey *et al.,* 2020 (*49*)	50 adults without DM with IDA	5.92 ± 0.37%	5.49 ± 0.42%	< 0.001	Decreased in adults without DM
Akkermans *et al.,* 2018 (*54*)	13 children and adolescents with T1DM with LID	NI	Mean HbA1c difference= -0.12 ± 0.58%	0.611	No change in children and adolescents with T1DM
Eser *et al.,* 2018 (*55*)	37 pregnant women without DM with IDA	5.01 ± 0.39%	4.69 ± 0.38%	< 0.05	Decreased in pregnant women without DM
Madhu *et al.*, 2017 (*59*)	60 adults without DM with IDA	5.5 ± 0.7%	5.0 ± 0.6% (decrease of 0.47%)	< 0.05	Decreased in adults without DM
Nasli-Esfagani *et al.,* 2017 (*60*)	45 adults with T2DM with IDA who received iron supplementation and 45 who received placebo	Iron supplementation: 7.59 ± 1.16% Placebo: 7.40 ± 1.01%	Iron supplementation: 6.80 ± 0.85% Placebo: 7.14 ± 0.95%	< 0.001 < 0.001	Decreased in adults with T2DM (iron supplementation reduced more HbA1c than placebo, p = 0.005)
Sinha *et al.*, 2012 (*68*)	50 adults and adolescents without DM with IDA	4.6%	5.9%	< 0.001	Increased in adults and adolescents without DM
Coban *et al.,* 2004 (*72*)	50 adults without DM with IDA	7.4 ± 0.8%	6.2 ± 0.6%	0.001	Decreased in adults without DM
Tarim *et al.*, 1999 (*73*)	11 adults with T1DM with ID and 11 adults without DM with ID	9,15 ± 2,47% WithT1DM: 10.6 ± 2.6% Without DM: 7.7 ± 1.3%	7,35 ± 2,27% WithT1DM: 8.3 ± 2.6% Without DM: 6.4 ± 1.2%	< 0.001 < 0.05 < 0.05	Decreased in adults with T1DM and without DM
DM - diabetes mellitus. Hb - hemoglobin. HbA1c - glycated hemoglobin. ID - iron deficiency. IDA - iron deficiency anemia. LID - latent iron deficiency. T1DM - type 1 diabetes mellitus. T2DM - type 2 diabetes mellitus. P ≤ 0.05 was considered statistically significant.					

Table 5 shows sample size and characteristics of patients, main results, and interference of IDA or LID in the HbA1c test in the studies included in the systematic review that evaluated HbA1c concentrations in patients with and without IDA or LID.

**Table 5:** Sample size and characteristics of patients, main results, and interference of iron deficiency anemia or latent iron deficiency in the HbA1c test of the studies included in the systematic review that evaluated the HbA1c concentrations of patients with and without iron deficiency anemia or latent iron deficiency

**Author, year**	**Sample size and characteristics of patients**	**HbA1c of IDA/LID/ID group**	**HbA1c of control group**	**P**	**Interference in HbA1c testing**
Tedong *et al*., 2026 (*15*)	135 adults with DM with IDA 75 adults with DM without IDA	9.11 ± 2.72%	9.08 ± 2.05%	0.91	IDA did not alter HbA1c in adults with DM
Deepali *et al*., 2025 (*16*)	50 adults without DM with IDA 50 adults without DM without IDA	6.1 ± 0.4%	5.7 ± 0.3%	< 0.05	IDA increased HbA1c in adults without DM
Koga and Ishibashi, 2025 (*17*)	15 adults with IDA 12 adults without IDA	6.2% (5.3–9.1)	7.8% (7.2–8.4)	0.157	IDA did not alter HbA1c in adults
Dutta *et al*., 2024 (*18*)	60 adults without DM with IDA 60 adults without DM without IDA	6.5 ± 0.5%	5.13 ± 0.8%	<0.001	IDA increased HbA1c in adults
Firat *et al*., 2024 (*19*)	240 pregnant women with DM (67 with IDA and 173 without IDA) 430 pregnant women without DM (103 with IDA and 327 without IDA)	5.67 ± 1.03% With DM: 6.4 ± 0.7% Without DM: 5.2 ± 0.9%	5.41 ± 0.83% With DM 6.0 ± 0.4% Without DM: 5.1 ± 0.8%	< 0.010 < 0.010 > 0.05	IDA increased HbA1c in pregnant women with DM, but not in those without DM
Haseena *et al*., 2024 (*20*)	50 pregnant women without DM with IDA 50 pregnant women without DM without IDA	6.21 ± 0.36%	4.54 ± 0.66%	< 0.001	IDA increased HbA1c in pregnant women without DM
Kanojia *et al*., 2024 (*21*)	50 adults without DM with IDA 50 adults without DM without IDA	7.16 ± 0.88%	5.49 ± 0.27%	< 0.001	IDA increased HbA1c in adults without DM
Kesharwani *et al*., 2024 (*22*)	100 adults without DM with IDA 100 adults without DM without IDA	6.0 ± 0.5%	5.4 ± 0.4%	< 0.001	IDA increased HbA1c in adults without DM
Krishnan *et al*., 2024 (*23*)	50 adults without DM with IDA 50 adults without DM without IDA	4.67 ± 0.39%	5.42 ± 0.29%	< 0.001	IDA decreased HbA1c in adults without DM
Kumar *et al*., 2024 (*24*)	60 adults without DM with IDA and without IDA	6.8 ± 1.1%	5.6 ± 0.9%	0.003	IDA increased HbA1c in adults without DM
Nakum *et al*., 2024 (*25*)	88 adults with T2DM with IDA 88 adults with T2DM without IDA	6.17 ± 1.58%	7.75 ± 1.82%	< 0.001	IDA decreased HbA1c in adults with T2DM
Suvethasri *et al*., 2024 (*27*)	100 adults without DM with IDA 100 adults without DM without IDA	5.87 ± 1.25%	4.97 ± 1.59%	< 0.001	IDA increased HbA1c in adults without DM
Shubham *et al*., 2024 (*28*)	50 adults without DM with IDA 50 adults without DM without IDA	5.92 ± 0.37%	5.11 ± 0.3%	< 0.001	IDA increased HbA1c in adults without DM
Thakker *et al*., 2024 (*30*)	155 pregnant women without DM with IDA (120 mild IDA x 30 moderate IDA x 5 severe IDA) 155 pregnant women without DM without IDA	5.44 ± 0.65% Moderate IDA: 6.24% Severe IDA: 6.52%	4.87 ± 0.42% Mild IDA: 5.31% Mild IDA: 5.31%	< 0.010 < 0.001 < 0.001	IDA increased HbA1c in pregnant women without DM, and the worsening of IDA severity increased HbA1c
Alzahani *et al.,* 2023 (*31*)	103 adults with IDA 104 adults without IDA	5.75 ± 0.35%	5.32 ± 0.48%	0.001	IDA increased HbA1c in adults
Elsheikh *et al*., 2023 (*32*)	41 with DM (11 with IDA and 30 without IDA) 404 without DM (130 with IDA and 274 without IDA)	With DM: 7.9% Without DM: 5.46%	With DM: 6.91% Without DM: 5.1%	< 0.001 0.249	IDA increased HbA1c in adults with DM, but not in adults without DM
Gharde *et al*., 2023 (*33*)	55 adults without DM with IDA 56 adults without DM without IDA	6.04 ± 0.74%	4.91 ± 0.65%	< 0.001	IDA increased HbA1c in adults without DM
Janice *et al*., 2022 (*39*)	31 women with moderate IDA 8 women with severe IDA	Severe IDA: 4.50 ± 0.34%	Moderate IDA: 5.18 ± 0.35%	< 0.001	Greater severity of IDA increased HbA1c in women
Jyothsna *et al.,* 2022 (*40*)	30 adults without DM with IDA 30 adults without DM without IDA	5.8 ± 0.40%	4.9 ± 0.55%	< 0.001	IDA increased HbA1c in adults without DM
Rao *et al.,* 2022 (*42*)	12,320 adults with IDA 21,440 adults without IDA	Men 5.7% Women: 6.0%	Men: 5.4% Women: 5.6%	< 0.001	IDA increased HbA1c in both men and women
Altuntas *et al*., 2021 (*43*)	131 adults without DM with IDA (29 mild IDA x 31 moderate IDA x 2 severe IDA) 132 adults without DM without IDA	5.4 ± 0.5%	5.9 ± 0.5% HbA1c did not alter as the severity of anemia worsened	< 0.001 > 0.05	IDA decreased HbA1c in adults without DM
Bindayel, 2021 (*44*)	21 women aged 20 to 50 years without DM with IDA (9 mild IDA x 12 moderate and severe IDA) 38 women aged 20 to 50 years without DM without IDA	5,40 ± 0,36% Mild IDA: 5.39 ± 0.38% Moderate and severe IDA: 5.40 ± 0.35%	Without IDA 5.15 ± 0.36%	> 0.05	IDA did not alter HbA1c in women < 50 years of age without DM
Kanchana and Pushpa, 2021 (*45*)	50 adults without DM with IDA 50 adults without DM without IDA	7.4 ± 0.5%	5.2 ± 0.8%	< 0.001	IDA increased HbA1c in adults without DM
Mahgoob and Moussa, 2020 (*47*)	72 children with T1DM with IDA 75 children with T1DM without IDA	8.04 ± 1.59%	7.21 ± 1.47%	< 0.001	IDA increased HbA1c in children with T1DM
Purbey *et al.,* 2020 (*49*)	50 adults without DM with IDA 50 adults without DM without IDA	5.92 ± 0.37%	5.11 ± 0.2%	< 0.001	IDA increased HbA1c in adults without DM
Intra *et al.,* 2019 (*51*)	86 adults and adolescents without DM with IDA 2,745 adults and adolescents without DM without IDA	5.59%	5.34%	< 0.001	IDA increased HbA1c in adults and adolescents without DM
Silva *et al.,* 2019 (*52*)	61 adults without DM with IDA (19 mild IDA x 26 moderate IDA x 16 severe IDA) 61 adults without DM without IDA	IDA: 5.6 ± 0.4% (HPLC) and 5.7 ± 0.4% Mild IDA: 5.5 ± 0.4% (HPLC) and 5.5 ± 0.3% (turbidimetry) Moderate IDA: 5.6 ± 0.4% (HPLC) and 5.7 ± 0.4% (turbidimetry) Severe IDA: 5.7 ± 0.4% (HPLC) and 5.9 ± 0.4% (turbidimetry)	5.3 ± 0.4% (HPLC) and 5.3 ± 0.3% (turbidimetry)	< 0.001 < 0.001 > 0.05 > 0.05	IDA increased HbA1c in adults without DM
Hashimoto and Koga, 2018 (56)	42 pregnant women with IDA and without IDA 42 pregnant and nonpregnant women with IDA and without IDA	NI NI	NI NI	< 0.001 > 0.05	IDA increased HbA1c in pregnant women, but not in nonpregnant women
Intra *et al*., 2018 (*57*)	109 adults and adolescents without DM with IDA 2,516 adults and adolescents without DM without IDA	5.53%	5.34%	< 0.001	IDA increased HbA1c in adults without DM
Madhu *et al.*, 2017 (*59*)	62 adults without DM with IDA 60 adults without DM without IDA	5.5 ± 0.7%	4.9 ± 0.5%	< 0.001	IDA increased HbA1c in adults without DM
Rajagopal *et al.,* 2017 (*61*)	75 adults without DM with IDA (40 mild IDA x 30 moderate IDA x 5 severe IDA) 75 adults without DM without IDA	6.84 ± 0.07%	5.12 ± 0.04% HbA1c increased as the severity of anemia worsened	< 0.001 < 0.05	IDA increased HbA1c concentrations in adults
Hong *et al.,* 2015 (*63*)	476 adults with IDA 414 adults without IDA Normoglycemia (FPG < 100 mg/dL) Prediabetes (100 ≤ FPG < 126 mg/dL) DM (FPG ≥ 126 mg/dL)	5.70 ± 0.02% 5.59 ± 0.02% 6.00 ± 0.05% 7.40 ± 0.46%	5.44 ± 0.03% 5.40 ± 0.03% 5.75 ± 0.06% 6.81 ± 0.23%	< 0.001 < 0.001 0.005 0.003	IDA increased HbA1c in adults with normoglycemia, prediabetes and DM
Christy *et al.,* 2014 (*64*)	120 adults (70 women and 50 men) without DM with IDA and without IDA Normoglycemia (FPG < 100 mg/dL) Prediabetes (100 ≤ FPG < 126 mg/dL)	6.87 ± 1.4% Women: 7.02 ± 1.58% Men: 6.67 ± 1.06% Normoglycemia: 6.43 ± 1.07% Prediabetes: 7.33 ± 1.55% Normoglycemia: 6.47 ± 1.19% (women) and 6.36 ± 0.88% (men) Prediabetes: 7.63 ± 1.76% (women) and 6.95 ± 1.15% (men)	5.65 ± 0.69% Women: 5.82 ± 0.53% Men: 5.59 ± 0.85% Normoglycemia: 5.46 ± 0.62% Prediabetes: 5.89 ± 0.75% Normoglycemia: 5.61 ± 0.58% (women) and 5.45 ± 0.67% (men) Prediabetes: 5.96 ± 0.46% (women) and 5.83 ± 1.00% (men)	< 0.05 < 0.05 < 0.05 < 0.05 < 0.05 < 0.05 < 0.05 < 0.05 < 0.05	IDA increased HbA1c concentrations in women and men with normoglycemia and prediabetes
Shanthi *et al.*, 2013 (*8*)	50 adults without DM with IDA 50 adults without DM without IDA	7.6 ± 0.5%	5.5 ± 0.8%	< 0.001	IDA increased HbA1c in adults without DM
Rafat *et al.,* 2012 (*66*)	30 pregnant women without DM with IDA 90 pregnant women without DM without IDA (30 in the first, 30 in the second, 30 in the third trimester)	5.19 ± 0.32%	4,58 ± 0.33% 4.56 ± 0.36% (first trimester) 4.48 ± 0.34% (second trimester) 4.69 ± 0.28% (third trimester)	< 0.001 < 0.001 < 0.001 < 0.001	IDA increased HbA1c in pregnant women without DM
Satriawibawa *et al.,* 2012 (*67*)	6 children and adolescents with T1DM with IDA 27 children and adolescents with T1DM without IDA	11.3 ± 2.5%	10.2 ± 2.4%	0.373	IDA did not alter HbA1c in children and adolescents with T1DM
Sinha *et al.*, 2012 (*68*)	50 adults and adolescents without DM with IDA 50 adults and adolescents without DM without IDA	4.6%	5.5%	< 0.001	IDA decreased HbA1c in adults and adolescents
Coban *et al.,* 2004 (*72*)	50 adults without DM with IDA 50 adults without DM without IDA	NI	NI	< 0.001	IDA increased HbA1c in adults
Pradeepa *et al.,* 2022 (*41*)	447 adults with T2DM with IDA 123 adults with T2DM with LID 117 adults with T2DM without IDA/LID	LID: 9.4 ± 2.0% IDA: 9.1 ± 1.7%	8.7 ± 0.8%	< 0.001 < 0.001	IDA and LID increased HbA1c in adults with T2DM
Akkermans *et al.,* 2018 (*54*)	13 children and adolescents with T1DM with absolute LID 211 without absolute LID 100 children and adolescents with T1DM with functional LID 104 without functional LID 7 children and adolescents with T1DM with IDA and 220 without IDA	Absolute LID: 7.7 ± 3.1% Funcional LID: 8.2 ± 3.6% IDA: 8.2 ± 3.1%	Without absolute LID: 8.1 ± 3.7% Without funcional LID: 8.1 ± 3.7% Without IDA: 8.2 ± 3.7%	0.360 0.618 0.947	IDA and LID did not alter HbA1c in children and adolescents with T1DM
Urrechaga, 2018 (*58*)	136 adults with T2DM with IDA 157 adults with T2DM with LID 368 adults with T2DM without LID/IDA M > 50 and < 50 years and F > 50 and < 50 years FPG < 7.0 mmol/L and > 7.0 mmol/L	IDA (women ≤ 50 years and FPG < 7.0 mmol/L): 6.4 ± 1.4% IDA (women > 50 years old and FPG < 7.0 mmol/L): 7.0 ± 1.2% IDA (women ≤ 50 years old and FPG > 7.0 mmol/L): 7.5 ± 1.5% IDA (women > 50 years old and FPG > 7.0 mmol/L): 8.5 ± 1.5% LID (women), IDA (men), LID (men)	Women ≤ 50 years and FPG < 7.0 mmol/L: 5.5 ± 0.9% Women > 50 years old and FPG < 7.0 mmol/L: 6.0 ± 0.8% Women ≤ 50 years old and FPG > 7.0 mmol/L: 6.6 ± 1.2% Women > 50 years old and FPG > 7.0 mmol/L: 7.8 ± 1.3% Without LID/IDA (women and men)	0.005 < 0.001 < 0.001 0.004 > 0.05	IDA increased HbA1c in women with T2DM, but not in men with T2DM LID did not alter HbA1c in women or men with T2DM
Ford *et al.,* 2011 (*69*)	818 adults with DM with LID, with IDA, and without LID/IDA 7,478 adults without DM with LID, with IDA, and without LID/IDA	With DM: 7.85% (IDA) With DM: 7.54% (LID) Without DM: 5.30% (IDA) Without DM: 5.39% (LID)	With DM: 7.71% Without DM: 5.31%	> 0.05 > 0.05	IDA and LID did not alter HbA1c in adults with and without DM
Koga *et al.,* 2010 (*71*)	50 women aged 20 to 50 years with LID 17 women aged 20 to 50 years with IDA 57 women aged 20 to 50 years without LID/IDA	IDA: 5.1 ± 0.2% LID: 5.0 ± 0.2%	4.8 ± 0.2%	< 0.001 < 0.05	IDA and LID increased HbA1c in women < 50 years of age
Mukasa et al., 2026 (*14*)	43 adults with T2DM with LID 103 adults with T2DM without LID	7.82% (6.36-9.74)	8.46% (6.82-9.93)	> 0.05	LID did not alter HbA1c in adults with T2DM
Kumar *et al.,* 2023 (*24*)	230 adults with and without ID	5.89 ± 0.43%	5.52 ± 0.50%	< 0.001	ID increased HbA1c in adults
Lyons *et al.,* 2020 (*46*)	520 women with ID and 1,612 without ID 68 men with ID and 1,177 without ID	5.61 ± 0.53% 5.6 ± 0.5% (women) 5.7 ± 0.7% (men)	5.50 ± 0.64% 5.5 ± 0.6% (women) 5.5 ± 0.7% (men)	< 0.001 0.002 0.088	ID increased HbA1c in women, but not in men
Sabitha *et al.,* 2020 (*50*)	53 adults with DM and good glycemic control (33 with LID and 20 without LID) 33 with DM and poor glycemic control (20 with LID and 13 without LID) 50 without DM (10 with LID and 40 without LID)	6.80 ± 1.82% With DM: 8.74 ± 1.82% DM (poor glycemic control): 9.90 ± 1.10% DM (good glycemic control): 6.82 ± 0.63% Without DM: 5.46 ± 0.38%	6.40 ± 1.33% With DM: 8.07 ± 1.25% DM (poor glycemic control): 8.90 ± 0.98% DM (good glycemic control): 6.80 ± 0.44% Without DM: 5.38 ± 0.49%	< 0.01 0.04 0.03 0.92 0.63	ID increased HbA1c in patients with DM with poor glycemic control, but not in patients with DM with good glycemic control or without DM
Tarim *et al.*, 1999 (*73*)	37 adults with T1DM (11 with ID and 26 without ID) 31 adults without T1DM (11 with ID and 20 without ID)	9.15 ± 2.41% With T1DM: 10.6 ± 2.6% Without T1DM: 7.7 ± 1.3%	8.39 ± 3.82% With T1DM: 10.3 ± 3.9% Without T1DM: 5.9 ± 1.3%	0.05 0.05 0.05	ID increased HbA1c in adults without T1DM, but not in those with T1DM
DM - diabetes mellitus. F - female. FPG - fasting plasma glucose. GDM - gestational diabetes mellitus. Hb - hemoglobin. HbA1c - glycated hemoglobin. ID - iron deficiency. IDA - iron deficiency anemia. LID - latent iron deficiency. M - male. NI - not informed. TS - transferrin saturation. TIBC - total iron-binding capacity. T1DM - type 1 diabetes mellitus. T2DM - type 2 diabetes mellitus. FPG - fasting plasma glucose. P ≤ 0.05 was considered statistically significant.					

Among the 43 studies evaluating the interference of IDA on HbA1c, most included adults of both sexes (31/43), with smaller proportions assessing pregnant women (5/43), women only (3/43), children and adolescents (3/43), or adults and adolescents (1/43). Diabetes status varied, with some studies including patients with T1DM (3/43), T2DM (3/43), or unspecified DM (5/43). Overall, IDA was associated with increased HbA1c concentrations in the majority of studies (33/43), while a few reported decreased (4/43) or unchanged values (6/43). Additional findings indicated that greater anemia severity was linked to higher HbA1c, and that the effect of IDA on HbA1c may vary according to factors such as pregnancy status, glycemic status and sex.

Among the 10 studies evaluating the effect of LID (without anemia) (6/10) or iron deficiency (with or without anemia) (4/10) on HbA1c, most included adults of both sexes (8/10), with smaller proportions assessing women only (1/10), and children and adolescents (1/10). Diabetes status varied, with some studies including patients with T2DM (3/10), T1DM (2/10), and unspecified DM (2/10). Overall, LID was associated with increased HbA1c concentrations in two studies (2/6), whereas four studies (4/6) reported no significant effect. In contrast, iron deficiency was consistently associated with increased HbA1c concentrations across all four studies. Notably, the effect of LID and iron deficiency on HbA1c may vary according to factors such as glycemic status and sex.

Table 6 shows sample size and characteristics of patients, main results, and interference in the risk of classification as prediabetes or DM by HbA1c test in the studies included in the systematic review that assessed this risk.

**Table 6:** Sample size and characteristics of patients, main results, and interference in the risk of classification as prediabetes or diabetes mellitus by HbA1c testing in the studies included in the systematic review that assessed this risk

**Author, year**	**Sample size and characteristics of patients**	**Main results**	**Interference in the risk of classification as prediabetes and DM by HbA1c test**
Dutta *et al*., 2024 (*18*)	60 adults without DM with IDA and without IDA	Each unit decrease in Hb increased the likelihood of HbA1c values in the prediabetic and diabetic range by 2.61 (1.65-3.50) times and 2.40 (1.81-3.77) times, respectively (P < 0.001) Each unit decrease in serum ferritin increased the likelihood of HbA1c values in the prediabetic and diabetic range by 1.02 (1.01-1.04) times and 1.01 (1.01-1.05) times, respectively (P < 0.001)	IDA increased the risk of classification as prediabetes and DM in adults
Estrella *et al*., _2022 (_*_38_*_)_	12,151 adults with DM, prediabetes and normoglycemia with LID, with IDA, and without LID/IDA	Women with IDA had a higher risk of classification as prediabetes (OR = 2.35 (1.68-3.28)) and DM [OR = 3.60 (1.60-8.08)] by HbA1c The increased risk of classification as prediabetes and DM was not observed in women with LID or in men with IDA or LID	IDA increased the risk of classification as prediabetes and DM by HbA1c in women, but not in men LID did not alter the risk of classification as prediabetes and DM by HbA1c in women or men
Lyons *et al.,* 2020 (*46*)	3,088 adults with DM or normoglycemia with and without LID	Women with ID had a higher risk of HbA1c ≥ 5.5% to <6.5% than women without ID [OR: 1.43 (1.08-1.87), P < 0.01] There was no association between ID and increased risk of HbA1c ≥ 5.5% to <6.5% in men, and there was no association between ID and increased risk of HbA1c ≥ 6.5% in men or women	ID increased the risk of classification as prediabetes by HbA1c in women, but not in men ID did not alter the risk of classification as DM by HbA1c in women or men
Purbey *et al.,* 2020 (*49*)	50 adults with prediabetes or normoglycemia with IDA before and after iron supplementation 50 adults with prediabetes or normoglycemia without IDA	The proportion of patients with prediabetes by HbA1c was higher in those with IDA (80%) than in those without IDA (0%) (P < 0.001) In the group of patients with IDA, the proportion of prediabetic patients decreased from 80% to 30% after iron supplementation (P < 0.001)	IDA increased the risk of classification as prediabetic by HbA1c in adults
Wei *et al.,* 2019 (*53*)	689 children and adolescents with DM or prediabetes or normoglycemia	Regression analysis demonstrated that transferrin concentrations and transferrin saturation were significantly associated with HbA1c concentrations (P < 0.05) The highest risk of HbA1c > 5.7% was found in the highest serum transferrin quartile and lowest transferrin saturation quartile in girls and boys	ID increased the risk of classification as DM or prediabetes by HbA1c in children and adolescents
Attard *et al.,* 2015 (*62*)	227 adults with DM or prediabetes or normoglycemia with LID, with IDA and without LID/IDA	The proportion of individuals diagnosed with DM by HbA1c was lower in those with LID (M: 12.9% and W: 19.1%) than in those without LID (M: 25.1% and W: 38.3%) (P < 0.05) The proportion of individuals diagnosed with prediabetes by HbA1c was higher in those with LID (H: 63% and M: 55.2%) than in those without LID (F: 42.6% and M: 49.0%) (P < 0.05) Women with LID had a lower relative risk of being classified as having DM by HbA1c than by FPG [RRR = 0.37 (0.15-0.88)] after adjusting for covariates Among women with LID, a lower percentage was predicted to have DM by HbA1c (0.5%) than by FPG (1.5%)	LID decreased the risk of classification as DM by HbA1c in women, but not in men
Hong *et al.,* 2015 (*63*)	9,775 adults with DM or normoglycemia with IDA and 476 without IDA	The weighted proportions of HbA1c concentrations ≥ 5.7% and ≥ 6.1% were higher in patients with IDA than in those without IDA after adjusting for confounding factors (P < 0.001 and P < 0.012, respectively) The weighted proportion of an HbA1c level ≥ 6.5% did not differ between patients with and without IDA after adjusting for confounding factors	IDA increased the risk of classification as prediabetes by HbA1c in adults IDA did not alter the risk of classification as DM by HbA1c in adults
Hardikar *et al.,* 2012 (*65*)	116 adults aged 21 years with DM or prediabetes or normoglycemia	The prevalence of prediabetes was 7.8% by OGTT and 23.3% by HbA1c, and the prevalence of DM was 2.6% by OGTT and 2.6% by HbA1c Twenty-four patients who were normoglycemic by OGTT were misclassified as having prediabetes or DM by HbA1c, and six patients with prediabetes or DM by OGTT were misclassified as normoglycemic by HbA1c Patients classified as having prediabetes or DM by HbA1c had lower ferritin concentrations (23.2 (6.6-46.7)) ng/mL] than those classified as normoglycemic by HbA1c (25.8 (7.9–53.8)) ng/mL]. Lower ferritin concentrations were significantly associated with higher HbA1c concentrations in the multivariate analysis	LID increased the risk of classification as prediabetes and DM by HbA1c in adults aged 21 years
Kim *et al.,* 2010 (*70*)	1,225 adults with DM or prediabetes or DM with ID and 9310 adults without ID	ID was associated with an increased risk of HbA1c ≥ 5.5% and ≥ 6.5% in women before and after adjustment for covariates ID was not associated with an increased risk of HbA1c ≥ 5.5% in men after adjusting for covariates The difference in the predicted prevalence of HbA1c ≥ 5.5% between women with and without ID was significant after adjusting for covariates (P < 0.05) The difference in the predicted prevalence of HbA1c ≥ 6.5% between women with and without ID and of HbA1c ≥ 5.5% between men with and without ID was not significant after adjusting for covariates	ID increased the risk of classification as prediabetes and DM by HbA1c in women, but not in men
DM - diabetes mellitus. F - female. HbA1c - glycated hemoglobin. ID - iron deficiency. IDA - iron deficiency anemia. LID - latent iron deficiency. M - male. FPG - fasting plasma glucose. OGTT - oral glucose tolerance test.			

Among these nine studies, most were conducted in adults of both sexes (8/9), with only one including children and adolescents. Overall, IDA was associated with an increased risk of HbA1c-based classification as prediabetes in all four studies that assessed this outcome and as DM in two of these studies. Latent iron deficiency was associated with an increased risk of classification as both prediabetes and DM in two studies (2/3). Similarly, iron deficiency was associated with an increased risk of classification as prediabetes in two studies (2/3) and as DM in one study (1/3). Some studies reported an increased risk limited to prediabetes, whereas others observed elevated risks for both prediabetes and DM, with certain findings suggesting sex-specific effects, particularly among women.

The results of the meta-analyses are shown in Figures 2A, 3A, and 4A and funnel plots in Figures 2B, 3B, and 4B. Fourteen studies were included in the meta-analysis that compared HbA1c concentrations before and after iron supplementation (Figure 2A). The random model was used to perform the meta-analysis, as the studies were heterogeneous (P < 0.001 and I^2^ = 99%). The meta-analysis showed that HbA1c concentrations decreased by an average of - 0.50% (- 0.92 to - 0.09) or 5.46 mmol/mol (- 10.05 to - 0.98) after iron supplementation (P = 0.02). HbA1c concentrations decreased after iron supplementation across multiple subgroup analyses (Table 7), including both sexes, pregnant women, studies including patients with DM, and those excluding individuals with CKD, anemia not caused by iron deficiency, or inflammation and/or elevated CRP, as well as when HbA1c was measured using HPLC and when anemia was defined according to the WHO (*12*). However, this effect was not observed in the subgroup in which HbA1c was measured by immunoturbidimetry, or in studies that excluded patients with DM.

**Figure 2:**
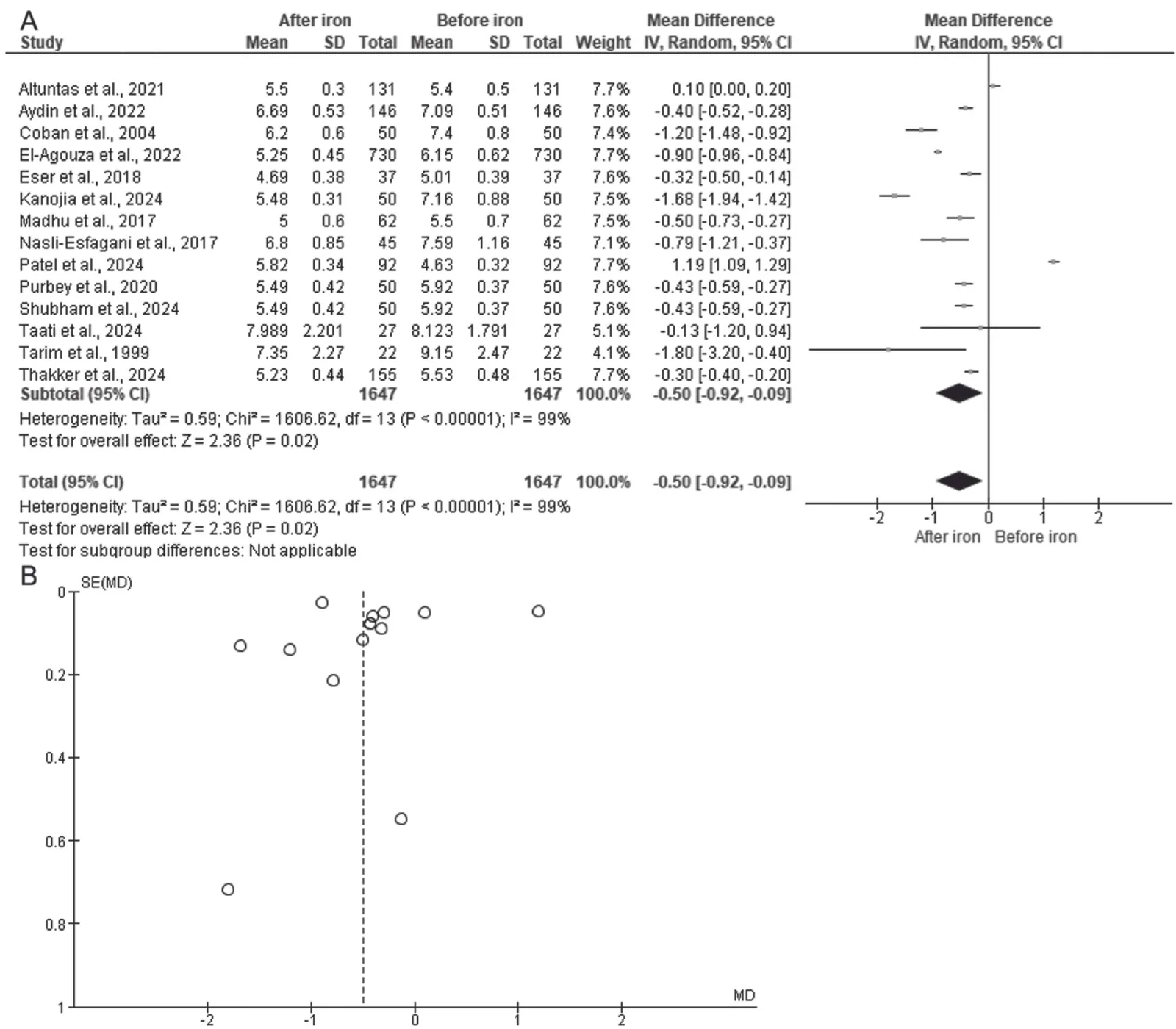
A) Meta-analysis of studies evaluating glycated hemoglobin (HbA1c) concentrations before and after iron supplementation. B) Funnel plot

**Figure 3:**
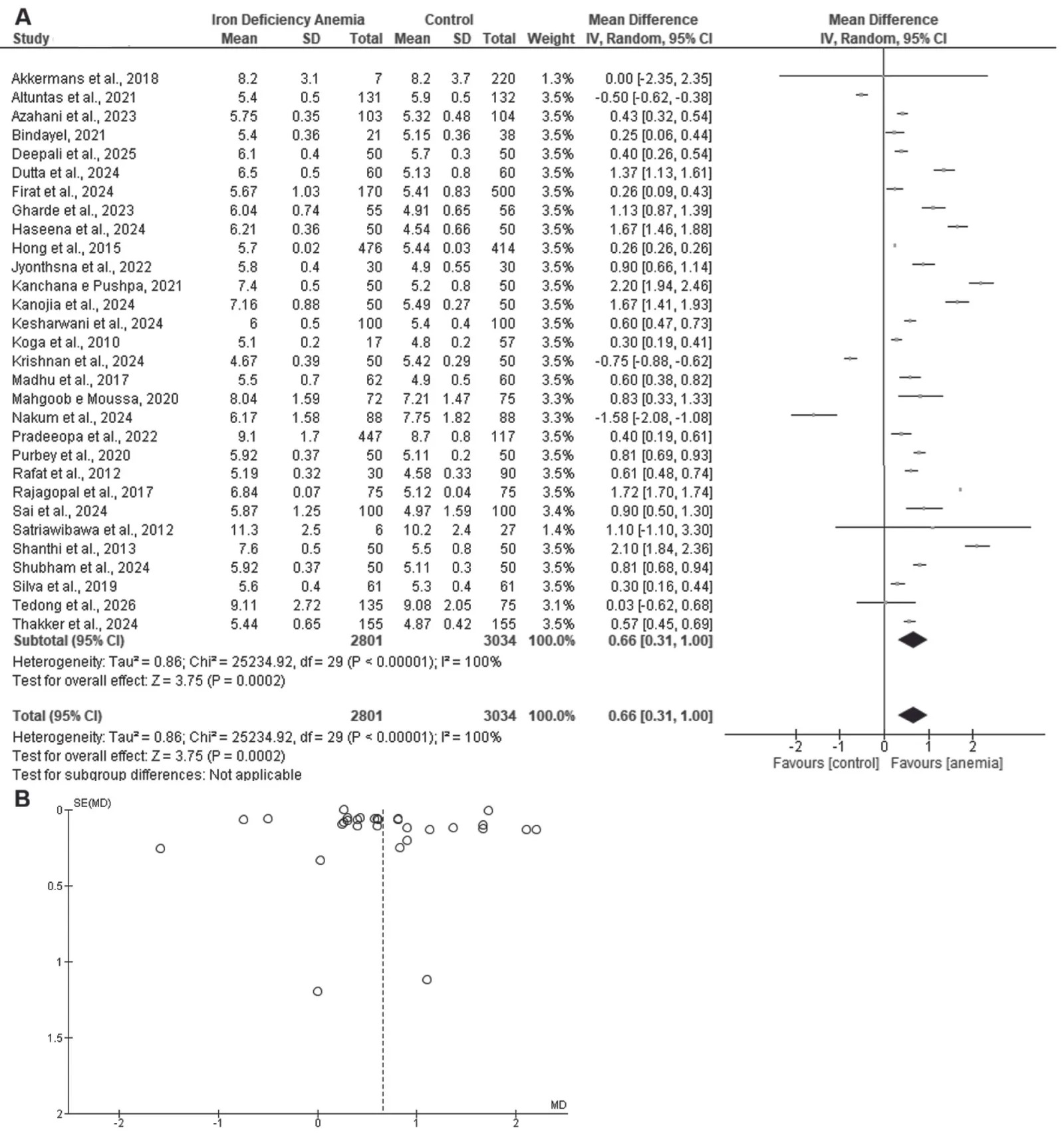
A) Meta-analysis of studies evaluating the interference of iron deficiency anemia in glycated hemoglobin (HbA1c) concentration. B) Funnel plot

**Figure 4:**
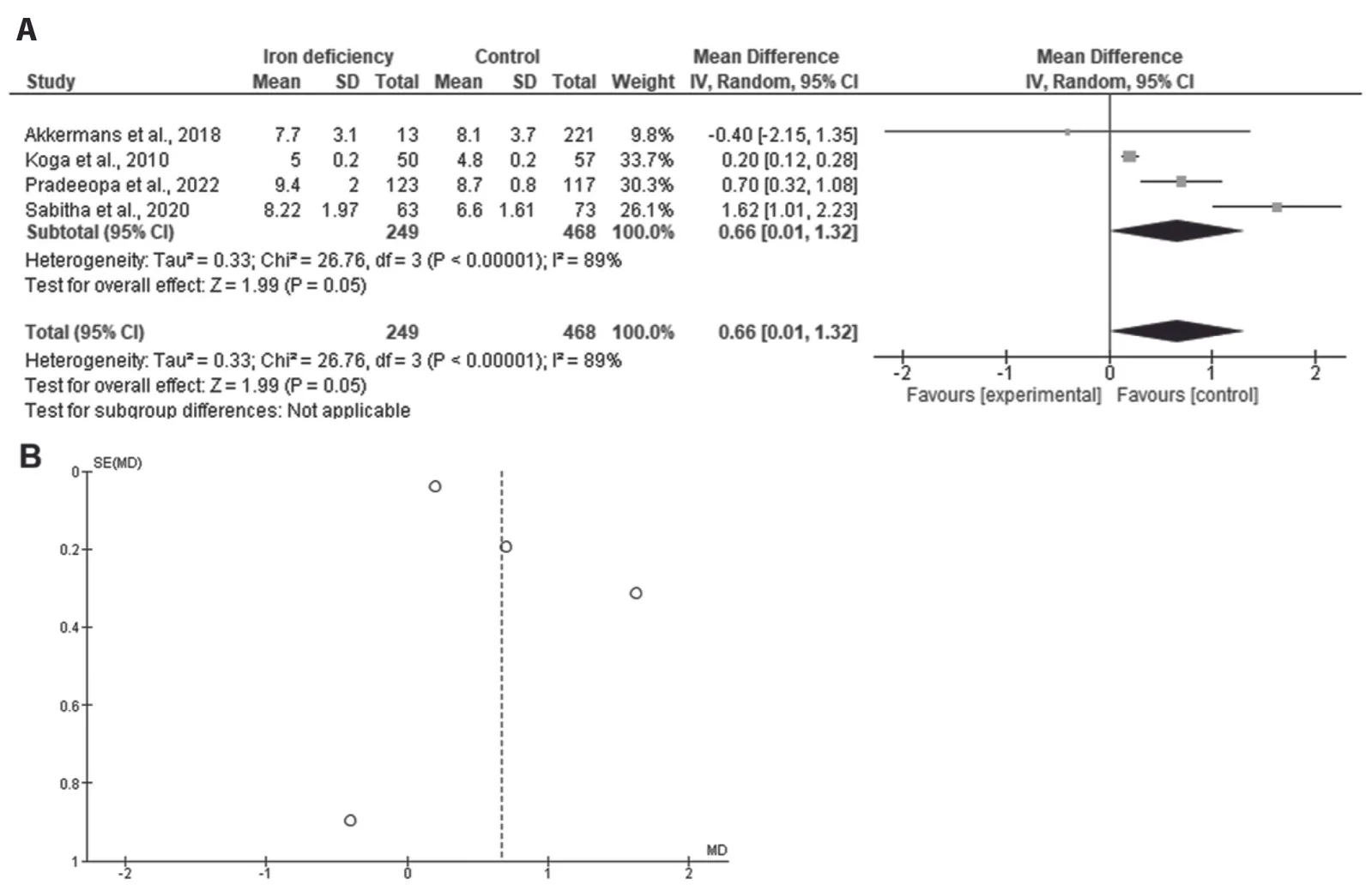
A) Meta-analysis of studies that evaluated the interference of latent iron deficiency on glycated hemoglobin (HbA1c) concentrations. B) Funnel plot

**Table 7:** Subgroup analysis of the meta-analysis that compared HbA1c concentrations before and after iron supplementation

**Subgroup**	**Mean difference of HbA1c** **(95% CI)**	**P**
Both sexes	- 0.54% (- 1.05 to - 0.04)	0.04
Pregnant women	- 0.31% (- 0.39 to - 0.22)	< 0.001
Only patients with DM	- 0.56% (- 0.94 to - 0.18)	0.004
Patients with DM were excluded	- 0.46% (- 1.02 to 0.09)	0.10
HbA1c was determined by HPLC	- 0.45% (- 0.81 to - 0.08)	0.02
HbA1c was determined by immunoturbidimetry	- 0.74% (- 1.62 to 0.14)	0.10
Patients with anemia not caused by iron deficiency were excluded	- 0.35% (- 0.52 to - 0.18)	< 0.001
Patients with CKD were excluded	- 0.36% (- 0.56 to - 0.17)	< 0.001
Patients with inflammation or elevated CRP were excluded	- 0.42% (- 0.53 to - 0.32)	< 0.001
Anemia was defined by Hb < 120 g/L (F) and < 130 g/L (M)	- 0.34% (- 0.68 to 0.00)	0.05
CI - confidence interval. CKD - chronic kidney disease. CRP - C-reactive protein. DM - diabetes mellitus. F - female. Hb - hemoglobin. HbA1c - glycated hemoglobin. HPLC - high performance liquid chromatography. M - male. P ≤ 0.05 was considered statistically significant.		

Thirty studies were included in the meta-analysis comparing HbA1c concentrations in patients with and without IDA (Figure 3A). A random model was used to perform the meta-analysis, as the studies were heterogeneous (P < 0.001 and I^2^ = 100%). The meta-analysis showed that HbA1c concentrations were higher in patients with IDA than in those without IDA, with a mean difference of 0.66% (0.31 to 1.00) or 7.21 mmol/mol (3.39 to 10.93) (P < 0.001). Concentrations of HbA1c were higher in patients with IDA compared with controls across several subgroup analyses (Table 8), including adults, children and adolescents, both sexes, nonpregnant women, pregnant women, and studies excluding individuals with DM, CKD, anemia not caused by iron deficiency, or inflammation and/or elevated CRP, as well as when HbA1c was measured by immunoturbidimetry. In contrast, this association was not observed in subgroups in which HbA1c was measured by HPLC, when iron deficiency and anemia were defined according to WHO criteria, or in studies including patients with DM (*12*, *13*).

**Table 8:** Subgroup analysis of the meta-analysis that compared HbA1c concentrations in patients with and without iron deficiency anemia

**Subgroup**	**Mean difference of HbA1c** **(95% CI)**	**P**
Adults	0.66% (0.31 to 1.01)	< 0.001
Children and adolescents	0.81% (0.34 to 1.28)	< 0.001
Both sexes	0.67% (0.27 to 1.06)	0.001
Nonpregnant women	0.29% (0.19 to 0.38)	< 0.001
Pregnant women	0.77% (0.30 to 1.24)	0.001
Only patients with DM	0.09% (- 0.45 to 0.62)	0.75
Patients with DM were excluded	0.85% (0.37 to 1.32)	< 0.001
HbA1c was determined by HPLC	0.45% (- 0.04 to 0.95)	0.07
HbA1c was determined by immunoturbidimetry	0.91% (0.17 to 1.65)	0.02
Patients with anemia not caused by iron deficiency were excluded	0.69% (0.32 to 1.06)	< 0.001
Patients with CKD were excluded	0.94% (0.53 to 1.34)	< 0.001
Patients with inflammation or elevated CRP were excluded	0.63% (0.33 to 0.93)	< 0.001
Anemia was defined by Hb < 120 g/L (F) and < 130 g/L (M)	0.49% (- 0.02 to 1.00)	0.06
Iron deficiency was defined by ferritin < 15 µg/L (≥ 5 years old) and < 12 µg/L (< 5 years old)	0.44% (- 0.17 to 1.05)	0.16
CKD - chronic kidney disease. CRP - C-reactive protein. DM - diabetes mellitus. F - female. Hb - hemoglobin. HbA1c - glycated hemoglobin. HPLC - high performance liquid chromatography. M - male. P ≤ 0.05 was considered statistically significant.		

Four studies were included in the meta-analysis comparing HbA1c concentrations in patients with and without LID (Figure 4A). The random model was used to perform the meta-analysis, as the studies were heterogeneous (P < 0.001 and I^2^ = 89%). The meta-analysis showed that HbA1c concentrations were higher in patients with LID or iron deficiency than in those without these conditions, with a mean difference of 0.66% (0.01 to 1.32) or 7.21 mmol/mol (0.11 to 14.43) (P = 0.05).

## Discussion

Iron deficiency anemia interferes with HbA1c measurement, leading to elevated HbA1c concentrations, as reported in most studies included in this systematic review, and corroborated by meta-analysis. Latent iron deficiency may also be associated with increased HbA1c concentrations, as indicated by meta-analysis (*8*, *16*, *18*-*22*, *24*, *27*, *28*, *30*-*33*, *39*-*42*, *45*, *47*, *49*, *51*, *52*, *56*-*59*, *61*, *63*, *64*, *66*, *71*, *72*). However, few studies evaluated this issue, and this finding was not consistently observed across the studies included in the systematic review, since only two of the six studies reported an association between LID and increased HbA1c concentrations (*41*, *71*). Furthermore, all studies that evaluated the interference of iron deficiency, regardless the presence of anemia, on HbA1c found increased HbA1c concentrations (*24*, *46*, *50*, *73*).

The elevation of HbA1c concentrations observed in patients with IDA or LID may be attributed to prolonged red blood cell survival. In iron deficiency, erythropoiesis is reduced, leading to an increased lifespan of circulating erythrocytes. Because the red blood cell membrane is highly permeable to glucose, this prolonged exposure enhances hemoglobin glycation, resulting in falsely elevated HbA1c concentrations (*6*, *74*). Additionally, iron deficiency may alter the structure of hemoglobin and promote hemoglobin peroxidation, accelerating hemoglobin glycation (*6*). This interference of IDA and LID on HbA1c concentrations was corroborated by studies showing that treatment of iron deficiency reduced HbA1c concentrations, a finding that was confirmed by meta-analysis (*21*, *28*, *30*, *31*, *35*-*37*, *48*, *49*, *55*, *59*, *60*, *72*, *73*).

Among the eleven studies included in this systematic review that evaluated the interference of IDA on HbA1c testing in patients with DM, six reported falsely elevated HbA1c concentrations (*19*, *32*, *41*, *47*, *58*, *63*). The remaining five studies did not report falsely elevated HbA1c concentrations (*15*, *25*, *54*, *60*, *67*). Among the four studies that evaluated the interference of LID on HbA1c testing in patients with DM, only one reported falsely elevated HbA1c concentrations (*41*). The remaining three studies did not report falsely elevated HbA1c concentrations (*14*, *54*, *58*). Furthermore, both studies that evaluated the interference of iron deficiency, regardless of the presence of anemia, on HbA1c testing in patients with DM found increased HbA1c concentrations (*50*, *73*). Therefore, while IDA appears to interfere with HbA1c test in patients with DM, leading to falsely elevated results, LID does not appear to result in significant interference in these patients. Some studies have also shown that the severity of IDA can intensify the false increase in HbA1c concentrations (*30*, *39*, *61*).

The false elevation of HbA1c caused by interference from IDA has important implications for clinical practice, as HbA1c values may not accurately represent actual glycemic status (*75*). The American Diabetes Association and the European Association for the Study of Diabetes recommend that HbA1c remain below the target of 7.0% for patients with T1DM and T2DM in order to avoid long-term complications (*76*). When HbA1c concentrations remain ≥ 7% despite treatment with oral antidiabetic drug or insulin, clinicians often intensify therapy by increasing dosages or introducing additional medications to achieve adequate glycemic control. However, in the presence of falsely elevated HbA1c values, this approach may lead to overtreatment and a higher risk of hypoglycemia (*75*). Therefore, HbA1c results in patients with DM and IDA should be interpreted with caution. In clinical scenarios where HbA1c reliability is compromised, alternative glycemic markers, such as self-monitoring of blood glucose, continuous glucose monitoring, fructosamine, or glycated albumin, are recommended (*77*).

Overestimation of HbA1c may also affect individuals without DM, leading to potential misdiagnosis of DM or prediabetes when values are falsely elevated due to IDA or LID. This issue was evaluated in nine studies (*18*, *38*, *46*, *49*, *53*, *62*, *63*, *65*, *70*). Among these, four studies reported that IDA increased the risk of classification as prediabetes (*18*, *38*, *49*, *63*). Two of these studies also reported an increased risk of classification as DM (*18*, *38*). Latent iron deficiency was associated with an increased risk of classification as both prediabetes and DM in one study (*65*). Iron deficiency, regardless of the presence of anemia, was associated with an increased risk of classification as prediabetes in three studies (*46*, *53*, *70*). Two of these studies also reported an increased risk of classification as DM (*53*, *70*). These findings indicate that HbA1c testing may lead to misdiagnosis of DM and prediabetes in individuals with IDA or LID. Given the high prevalence of these conditions, caution is warranted when interpreting HbA1c values. In patients with suspected iron deficiency, evaluation with a complete blood count to detect anemia, along with biomarkers of iron metabolism, preferably serum ferritin, should be considered. When iron deficiency is present, its correction and the use of alternative glycemic markers may be appropriate before establishing a definitive diagnosis (*7*, *8*). According to the American Diabetes Association, in clinical conditions associated with an altered relationship between HbA1c and glycemia, including disorders affecting red blood cell turnover, such as IDA, the diagnosis of DM should be based on plasma glucose criteria rather than HbA1c (*77*).

Furthermore, some studies have shown that IDA and LID increased the risk of classification as prediabetes and/or DM only in women, but not in men (*38*, *46*, *70*). In addition, two studies reported that women with iron deficiency had higher HbA1c concentrations compared to the control group, whereas no significant difference was observed among men (*46*, *58*). This may be explained by higher prevalence of IDA and LID in women, which is related to menstrual blood loss and nutritional deficiencies, particularly among those of reproductive age, and is associated with shifts in HbA1c distribution to higher concentrations (*38*, *46*, *70*).

Although most studies report falsely elevated HbA1c concentrations in IDA, some investigations have observed lower HbA1c values and increased concentrations after iron supplementation (*23*, *25*, *26*, *43*, *68*). These conflicting findings may be explained by differences in the diagnostic criteria used to define IDA, laboratory methods used for HbA1c measurement, and the presence of associated conditions, such as CKD, hemoglobinopathies, and other non-IDAs, which may reduce HbA1c concentrations, as well as inflammatory conditions, which may increase ferritin concentrations and interfere with the diagnosis of iron deficiency (*78*, *79*).

The assessment of publication bias, conducted through visual inspection of the funnel plots (Figures 2B, 3B, and 4B), demonstrated patterns of dispersion that suggest potential biases, with the analysis of Figure 4B being particularly limited by the small number of studies included in the meta-analysis of LID. This perception is corroborated by the methodological quality analysis, which revealed that most intervention studies (14/19) have a serious risk of bias according to the ROBINS-I V2 tool, while cross-sectional studies were mostly of moderate (24/42) or low (17/42) quality according to the NOS. Among the main factors that compromised the scientific rigor of the included studies were the absence of rigorous control for confounding variables, such as the presence of CKD and anemia not caused by iron deficiency, the non-strict adherence to the WHO diagnostic criteria for anemia and iron deficiency, and the use of laboratory methods for HbA1c without National Glycohemoglobin Standardization Program (NGSP) certification. These methodological weaknesses explain the high statistical heterogeneity detected in the meta-analyses and impose caution in generalizing the findings.

The American Diabetes Association recommends that HbA1c should be measured in a laboratory using methods certified by the NGSP and traceable to the International Federation of Clinical Chemistry (IFCC) reference system, ensuring alignment with the Diabetes Control and Complications Trial (DCCT) assay (*77*). However, the NGSP recognize that HbA1c results may be affected by biological factors, regardless of the analytical method used (*78*). Thirty-three studies used HPLC to measure HbA1c (*16*, *17*, *19*, *22*, *31*, *34*, *36*, *38*, *40*, *41*, *43*, *44*, *46*, *50*-*54*, *56*-*66*, *69*-*71*, *73*). However, only eight studies reported using an NGSP-certified method (*18*, *51*, *52*, *56*, *57*, *59*, *65*, *70*). Subgroup analysis suggested that immunoturbidimetric methods may be more susceptible to interference from IDA, resulting in more pronounced elevations in HbA1c compared to HPLC. One possible explanation is that turbidimetric methods rely on antibodies to recognize the glycated fraction of hemoglobin. In the presence of IDA, structural alterations in hemoglobin may promote cross-reactivity or exaggerated antibody binding, potentially leading to falsely elevated results. In contrast, HPLC separates hemoglobin fractions based on ionic charge, which may make it less sensitive to certain structural changes that do not significantly affect the isoelectric point of the molecule (*6*, *80*).

According to the WHO, anemia is defined by hemoglobin concentrations < 120 g/L in nonpregnant women and < 130 g/L in men aged 15-65 years, with specific reference values also established for children and pregnant women (*12*). Iron deficiency is defined by serum ferritin concentrations < 12 µg/L in children under 5 years of age and < 15 µg/L in individuals aged 5 years or older (*13*). However, only a subset of the included studies applied these criteria for the diagnosis of anemia and/or iron deficiency, which contributed to the heterogeneity observed across studies (*15*, *18*, *19*, *22*-*24*, *28*, *29*, *31*-*33*, *36*, *41*, *43*, *44*, *46*-*49*, *51*, *52*, *54*-*56*, *58*-*63*, *65*, *68*, *71*). This finding was supported by subgroup analysis, which showed that the associations between IDA and increased HbA1c was not observed in studies that strictly applied the WHO criteria to define anemia and iron deficiency. This indicates that HbA1c elevation may be less consistent when more stringent diagnostic criteria are employed.

Another limitation of this systematic review is that exclusion criteria varied widely across the included studies. Several studies excluded patients with anemia not caused by iron deficiency, such as hemoglobinopathies, hemoglobin variants, thalassemia, hemolytic anemia, acute or chronic blood loss, which is important since these can result in falsely reduced HbA1c concentrations (*8*, *14*-*16*, *18*-*24*, *27*-*34*, *36*, *39*-*41*, *43*-*49*, *51*, *54*, *55*, *57*-*61*, *64*, *66*-*68*, *72*, *78*). Patients with CKD were also excluded in many studies, which is relevant because CKD may lead to anemia of chronic disease and falsely reduced HbA1c concentrations (*8*, *14*, *16*, *18*, *20*, *22*, *24*, *27*, *28*, *30*-*34*, *36*, *38*-*40*, *43*-*46*, *49*, *51*, *56*, *57*, *59*-*61*, *64*, *67*, *68*, *70*, *72*, *81*). Subgroup analysis demonstrated that IDA was associated with increased HbA1c concentrations, and iron supplementation was associated with a reduction in HbA1c concentrations in studies that excluded patients with CKD or anemia not caused by iron deficiency. However, these findings should be interpreted with caution, given the considerable heterogeneity across studies in the definition of anemia not caused by iron deficiency.

Some studies excluded patients with inflammatory or infectious conditions and/or elevated CRP concentrations (*15*, *19*, *36*, *40*, *50*, *51*, *54*, *56*-*60*, *67*). This is an important consideration, as ferritin is an acute-phase reactant, and its concentration increase in the presence of inflammation, potentially interfering with the diagnosis of IDA and LID when assessed by ferritin concentrations (*13*, *79*). Subgroup analysis demonstrated that IDA was associated with increased HbA1c concentrations, and iron supplementation was associated with a reduction in HbA1c concentrations in studies that excluded patients with inflammation and/or elevated CRP.

Subgroup analysis also showed that the association between IDA and elevated HbA1c remained significant across various population groups, including adults of both sexes, nonpregnant and pregnant women, children and adolescents, and individuals without DM. Therefore, the findings of this study consolidate and expand the evidence on the interference of iron deficiency in HbA1c measurement, demonstrating, based on a meta-analysis of 30 studies, that IDA is associated with a significant increase in HbA1c concentrations, in contrast to previous systematic reviews that included a smaller number of studies (*82*, *83*). Furthermore, these results corroborate prior evidence that iron supplementation reduces HbA1c concentrations (*5*, *84*). However, the methodological quality assessment and subgroup analyses indicate that these findings should be interpreted with caution, as the methods used to measure HbA1c and the diagnostic criteria for anemia and iron deficiency represent important sources of bias.

Some additional relevant aspects of this study include the analysis of the effect of LID on HbA1c concentrations, which may also be associated with increased HbA1c concentrations. However, this finding is limited by the small number of available studies. Another relevant aspect was the assessment of the risk of diagnostic error, suggesting that iron deficiency may increase the likelihood of misclassifying individuals as prediabetic or diabetic, although the current evidence remains inconclusive.

In conclusion, IDA interferes with HbA1c measurement, leading to falsely elevated HbA1c concentrations. This effect was observed in most of the studies included in the systematic review and was corroborated by the meta-analysis results. However, these findings should be interpreted with caution, particularly because the HbA1c assay used, including NGSP-certified HPLC methods, and the diagnostic criteria for anemia and iron deficiency, such as WHO definitions, may influence the magnitude of this effect. Latent iron deficiency may also be associated with increased HbA1c concentrations, as indicated by meta-analysis. However, this finding is limited by the small number of available studies.

Given the high prevalence of IDA and LID in the general population, it is important that HbA1c testing be accompanied by a complete blood count and assessment of iron status, preferably ferritin. This approach may help ensure a more accurate interpretation of HbA1c results in individuals with iron deficiency.

## APPENDIX

The articles were selected using the following combination of descriptors, which were defined according to Medical Subject Headings (MeSH): (“Glycated hemoglobin” or “Hemoglobin, Glycated” or “Glycohemoglobin” or “Glycohemoglobins” or “Glycated Hemoglobins” or “Hemoglobins, Glycated” or “Hemoglobin, Glycosylated” or “Glycosylated Hemoglobin” or “Glycated Hemoglobin A1c” or “Hemoglobin A1c, Glycated” or “Glycosylated Hemoglobin A1c” or “Hemoglobin A1c, Glycosylated” or “Hb A1a-2” or “Hemoglobin, Glycated A1a-2” or “A1a-2 Hemoglobin, Glycated” or “Glycated A1a-2 Hemoglobin” or “Hemoglobin, Glycated A1a 2” or “Glycated Hemoglobin A” or “Hemoglobin A, Glycated” or “Hb A1a+b” or “Hb A1c” or “HbA1” or “Glycosylated Hemoglobin A” or “Hemoglobin A, Glycosylated” or “Hb A1” or “Glycohemoglobin A” or “Hemoglobin A(1)” or “Hemoglobin, Glycosylated A1a-1” or “A1a-1 Hemoglobin, Glycosylated” or “Glycosylated A1a-1 Hemoglobin” or “Hemoglobin, Glycosylated A1a 1” or “Hb A1a-1” or “Hemoglobin, Glycated A1b” or “A1b Hemoglobin, Glycated” or “Glycated A1b Hemoglobin” or “Hb A1b” or “Hemoglobin, Glycosylated A1b” or “A1b Hemoglobin, Glycosylated” or “Glycosylated A1b Hemoglobin” or “Fructated Hemoglobins” or “Hemoglobins, Fructated”) and (“anemia, iron-deficiency” or “Anemia, Iron Deficiency” or “Iron-Deficiency Anemia” or “Iron Deficiency Anemia” or “Anemias, Iron-Deficiency” or “Anemias, Iron Deficiency” or “Iron-Deficiency Anemias” or “Iron Deficiency Anemias” or “Iron Deficiencies” or “Deficiencies, Iron” or “Deficiency, Iron” or “Sideropenia” or “Sideropenias” or “Iron Deficiency” or “Hypoferritinemia” or “Hypoferritinemias” or “Latent Iron Deficiency” or “Deficiencies, Latent Iron” or “Deficiency, Latent Iron” or “Iron Deficiencies, Latent” or “Iron Deficiency, Latent” or “Latent Iron Deficiencies”).

## Data Availability

The data generated and analyzed in the presented study are available from the corresponding author on request.
